# Evaluating MiRNAs in Blood-Based Liquid Biopsy for Early-Onset Colorectal Cancer Detection: A Systematic Review and Meta-Analysis

**DOI:** 10.3390/cancers18050720

**Published:** 2026-02-24

**Authors:** Aman Ullah, Mustafa Ghulam, Jiahao Liu, Sanfei Peng, Yuhan Yin, Sihan Wu, Yang Fu

**Affiliations:** 1 Department of Gastrointestinal Surgery, The First Affiliated Hospital of Zhengzhou University, Zhengzhou 450052, China; dramanullahkhanbalouch@gmail.com (A.U.); ljh13783593694@gs.zzu.edu.cn (J.L.); yuhan.yin@zzu.edu.cn (Y.Y.); sihanwu2025@gs.zzu.edu.cn (S.W.); 2 Department of Environmental Ecology and Landscape Management, Comenius University Bratislava, 84215 Bratislava, Slovakia; mustafa.ghulam@uniba.sk

**Keywords:** early-onset colorectal cancer, colorectal cancer, circulating microRNAs, liquid biopsy, blood-based biomarkers, diagnostic accuracy

## Abstract

**Background:** A significant rise is observed in the incidence of early-onset colorectal cancer (EOCRC) worldwide, demanding discovery of promising non-invasive diagnostic biomarkers. This systematic review and meta-analysis evaluated the diagnostic accuracy of single circulating microRNAs (miRNAs) for EOCRC detection. **Methods:** The protocol for this systematic review was prospectively registered with PROSPERO (registration number: CRD420251252155). We systematically searched PubMed, Embase, Web of Science and Scopus through September 2025 following PRISMA 2020 guidelines. Studies stating diagnostic accuracy of single circulating miRNAs in blood samples for histologically confirmed CRC were included. The quality assessment of included studies was done by using QUADAS-2 and bivariate random-effect meta-analysis was performed to calculate pooled diagnostic metrics. **Results:** Sixteen studies comprising 909 CRC cases and 1214 controls evaluating 22 distinct miRNAs were included. In the primary meta-analysis restricted to early-onset colorectal cancer (EOCRC, <50 years), pooled sensitivity was 84.4% and specificity was 85.7%. Analyses including mixed-age or all CRC populations were conducted as secondary analyses and showed comparable diagnostic performance. Subgroup analysis showed EOCRC patients (<50 years, n = 15) demonstrated sensitivity of 84.4% and specificity of 85.7%. Substantial heterogeneity existed (I^2^ > 85%). Quality assessment revealed high risk of bias in patient selection (87.5%) and index test domains (87.5%). Mechanistic analysis identified key pathways including Wnt/β-catenin, PI3K/AKT and EMT regulation. Sensitivity analyses confirmed robust estimates and Deeks’ test (*p* = 0.99) indicated no publication bias. **Conclusion:** This study has shown that individual circulating miRNAs provide consistent diagnostic accuracy for early-onset colorectal cancer (EOCRC); however, these findings require prospective validation in true screening settings before clinical implementation.

## Simple Summary

Colorectal cancer is increasingly being found in younger adults under age 50, highlighting a need for easier early detection methods. This research reviewed existing studies on a promising type of blood test that looks for tiny molecules called microRNAs, which are often released by cancer cells. Our goal was to assess how accurate these single-molecule blood tests are at spotting early-onset colorectal cancer. We found that, overall, these tests show high accuracy, meaning they may have potential relevance for future screening applications for younger people. While these results are encouraging for future cancer detection, more standardized and large-scale studies are needed to confirm their reliability before they can be used widely in clinics.

## 1. Introduction

Colorectal cancer (CRC) is a significant health burden, ranking as the third most frequent type of cancer and second leading cause of cancer-associated mortality globally, with 1.9 million new cases and 904,000 deaths in 2022 [1]. The incidence of early-onset colorectal cancer (EOCRC), diagnosed before age 50, is increasing globally at an alarming rate of 2–4% annually, and is projected to exceed 3.2 million cases by 2040, a predicted 63% rise [2]. The prognosis of patients with CRC varies markedly according to the severity of disease at the time of diagnosis. Localized CRC has a 90% survival rate, while CRC with metastasis has a survival rate of less than 15% [3]. Hence, early detection of CRC is crucial, especially in younger patients who are often excluded from routine screening.

The current diagnostic approaches for CRC, including colonoscopy, serum CEA levels and fecal-based tests such as FOBT and FIT, have certain limitations. Although colonoscopy is an effective approach, it is invasive and has poor patient compliance, especially in younger individuals [3]. The limitation of fecal-based tests (FOBT and FIT) lies in their inadequate sensitivity (40–70%) to detect early-stage disease [4], whereas serum CEA demonstrates sensitivity of 40% to 50% and limited specificity [5]. These limitations make the early detection of EOCRC challenging, where deferred diagnosis is frequent due to its atypical manifestation and minimal clinical suspicion. Consequently, there is an urgent need for sensitive, non-invasive and specific biomarkers [6].

MicroRNAs (miRNAs) are 19–25-nucleotide non-coding RNAs that regulate approximately 60% of human protein-coding genes through post-transcriptional silencing [7]. The discovery of stable, cell-free circulating miRNAs in body fluids has revolutionized cancer biomarker research [8]. Their stability results from packaging within extracellular vesicles or association with RNA-binding proteins, which protects them from RNase degradation [9]. Circulating miRNAs have great potential as biomarkers because of their remarkable stability in pre-analytical settings, specific expression patterns related to different cancer types, the ability to be detected through well-established quantitative methods (such as RT-qPCR and NGS), and the low invasiveness of blood collection, which allows for ongoing monitoring of patients [10].

Several circulating miRNAs have been investigated as potential biomarkers for the detection of CRC. According to meta-analyses, miR-21 demonstrates sensitivity of 65–75% and specificity of 80–90%, while miR-92a exhibits sensitivity of 70–80% and specificity of 75–85% for CRC diagnosis [11,12]. However, most studies have predominantly concentrated on average-risk or older populations, with limited investigation specifically in EOCRC cohorts. It remains unclear whether miRNA biomarkers validated in older populations will perform similarly in younger patients because EOCRC has distinct molecular and clinical characteristics, such as different mutational profiles and tumor features [13].

Knowledge about the molecular mechanisms through which miRNAs contribute to CRC pathogenesis provides essential advantages, which include rational candidate selection, augmented biological credibility and interpretation within the tumor biology context [14]. Contemporary studies have shed light on the regulatory functions of CRC-associated miRNAs. For instance, miR-21 targets tumor suppressor genes that include PTEN, PDCD4, and RECK, and activates PI3K/AKT signaling and promotes invasion, while miR-143 and miR-145 target KRAS, slowing MAPK pathway activation [15,16]. Insight into these mechanistic relationships confirms miRNAs as biologically relevant biomarkers and delivers understanding of molecular pathways which undergo dysregulation in EOCRC.

The majority of analyses have not been classified according to age of onset. The diagnostic accuracy across studies varies widely, in part due to differences in sample types, methods of detection, normalization techniques, and patient populations [17]. EOCRC has different characteristics, including more frequent occurrence of left-sided tumors and a different mutational pattern [18]. Distinct profiles of miRNAs might be found in EOCRC patients, and systematic evaluation is required to identify clinically relevant biomarkers. Several individual circulating miRNAs that have association with EOCRC also need to be systematically assessed for their potential as clinical diagnostic tools.

Recent developments have made it possible to identify opportunities and obstacles. A plasma signature of four miRNAs (miR-193a-5p, miR-210, miR-513a-5p, miR-628-3p) demonstrated strong performance, with an AUC of 0.92 in the discovery set and 0.88 in validation, and levels declined after surgery, indicating tumor specificity [19,20,21]. Nonetheless, results from these studies show considerable variation. A meta-analysis of 37 studies reported a pooled AUC of ~0.86 (sensitivity 0.76, specificity 0.83), indicating moderate diagnostic ability [22]. Another analysis of miRNA panels showed a higher AUC (~0.915); however, it noted differences between serum and plasma, methodological inconsistencies, and lack of standardized normalization [18]. Furthermore, results from studies performed using Mendelian randomization methods support the association of circulating miRNAs identified in plasma (e.g., miR-21-5p, miR-146a) with CRC risk and, consequently, indicating potential links to genetic susceptibility [23]. Collectively, circulating miRNAs may be a promising and non-invasive tool for detection of EOCRC, but researchers have faced many challenges including inter-study variability, limited standardization, and insufficient validation in younger and diverse populations. Therefore, future research should be focused on creating large, multi-ethnic EOCRC cohorts, methodological harmonization, and integration of genetic risk markers.

### Specific Study Objectives

The primary objective of this study is to evaluate the diagnostic accuracy of single circulating blood-based miRNAs for detecting colorectal cancer, with a focus on early-onset colorectal cancer (EOCRC, <50 years).

## 2. Methods

### 2.1. Protocol and Registration

**Figure 1:**
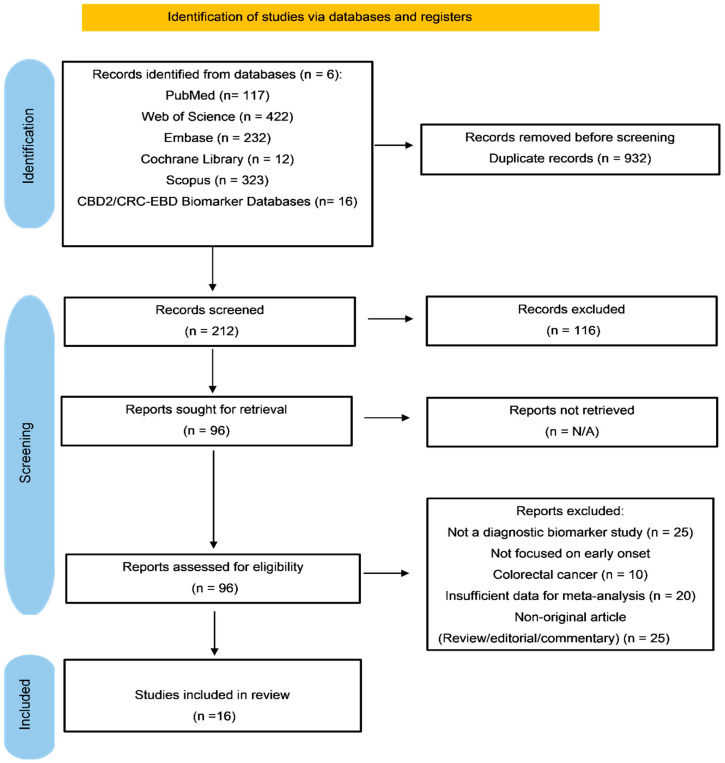
PRISMA flow diagram illustrating the study selection. The diagram illustrates the number of records identified, screened, excluded and included in the systematic review, in accordance with the Preferred Reporting Items for Systematic Reviews and Meta-Analysis (PRISMA) guidelines.

This systematic review was conducted according to PRISMA 2020 guidelines for diagnostic test accuracy studies [24]. The whole study selection method is presented in Figure 1. The protocol for this systematic review was prospectively registered with PROSPERO (registration number: CRD420251252155). The registered protocol is available at https://www.crd.york.ac.uk/PROSPERO/view/CRD420251252155 (accessed on 11 December 2025).

### 2.2. Search Strategy

We conducted a comprehensive literature search across PubMed/MEDLINE, Embase, Web of Science, Cochrane Library, and Scopus from inception to 1 September 2025. Supplementary searches included reference list screening, forward citation tracking, clinical trial registries (ClinicalTrials.gov, WHO ICTRP) and conference proceedings (ASCO, ESMO, AACR). The search combined three domains: disease terms (colorectal cancer, colon cancer, rectal cancer, CRC, early-onset colorectal cancer, EOCRC), biomarker terms (microRNA, miRNA, miR-, circulating RNA, cell-free RNA) and diagnostic terms (biomarker, diagnosis, screening, detection, sensitivity, specificity, ROC curve). Both controlled vocabulary (MeSH, Emtree) and free-text terms were used without language or date restrictions. Complete search strategies are in Supplementary Table S1.

### 2.3. Eligibility Criteria

We included studies evaluating adults (≥18 years) with histologically confirmed colorectal cancer (CRC), including early-onset colorectal cancer (EOCRC) diagnosed before age 50, compared against controls (healthy individuals or patients with benign colorectal conditions). Control populations varied across included studies and comprised either healthy individuals or patients with benign colorectal conditions. In several studies, control status was confirmed by colonoscopy, whereas in others controls were defined based on clinical assessment and absence of known colorectal disease. This variability reflects differences in study design and was considered when interpreting diagnostic accuracy. Given the limited availability of studies exclusively enrolling EOCRC populations, studies assessing mixed-age CRC cohorts were also eligible, provided that their design did not explicitly exclude patients under 50 years and that sufficient diagnostic accuracy data were reported.

Index tests comprised single circulating microRNA (miRNA) biomarkers measured in blood samples (serum, plasma, or whole blood). Studies were required to use histopathology and/or colonoscopy as the reference standard and to provide sufficient data to construct 2 × 2 contingency tables or report sensitivity and specificity with corresponding sample sizes. Studies were required to include at least 20 participants (CRC cases and controls combined). No minimum threshold for CRC cases alone was imposed, reflecting the limited size of early diagnostic studies in this field.

The primary outcomes were sensitivity, specificity, positive predictive value (PPV), negative predictive value (NPV), diagnostic odds ratio (DOR), likelihood ratios, and area under the curve (AUC). Secondary outcomes included stage-specific diagnostic accuracy and performance in age-stratified subgroups, with particular emphasis on EOCRC (<50 years).

Studies without explicit EOCRC stratification or with unclear age reporting were not excluded a priori but were addressed through predefined subgroup and sensitivity analyses to assess the robustness of EOCRC-specific findings.

We excluded animal or in vitro studies without human validation, reviews, meta-analyses, editorials, case reports, conference abstracts without full text, studies evaluating only tissue miRNAs, multi-miRNA panels without individual biomarker performance data, overlapping cohorts, and studies lacking sufficient data for diagnostic accuracy estimation.

### 2.4. Study Selection and Data Extraction

Two reviewers independently screened titles/abstracts and full texts, with disagreements resolved through discussion or third-party adjudication. Inter-rater agreement was assessed using Cohen’s kappa. Selection was documented using a PRISMA flow diagram. Data were independently extracted using standardized forms including study characteristics (author, year, country, design, sample sizes, funding), population details (age distribution, sex, stage distribution, tumor location, controls), miRNA details (specific miRNAs, sample type, collection/processing protocols, extraction method, detection method, normalization strategy, cut-off values), diagnostic accuracy data (TP, FP, TN, FN, sensitivity, specificity, AUC), mechanistic information (target genes, signaling pathways, functional validation), and quality indicators (blinding, prospective/retrospective design, consecutive enrollment).

### 2.5. Quality Assessment

Methodological quality was assessed using QUADAS-2, evaluating risk of bias and applicability across four domains: patient selection, index test, reference standard, and flow/timing [25]. Each domain was rated as low, high, or unclear risk. Studies with high risk in ≥2 domains were considered poor-quality. Two reviewers independently performed assessments with Cohen’s kappa for agreement.

### 2.6. Statistical Analysis

The primary meta-analysis was restricted to studies including early-onset colorectal cancer (EOCRC; <50 years). Meta-analyses including mixed-age or late-onset CRC populations were performed as secondary analyses for contextual comparison. An overall pooled analysis including all CRC populations was conducted as an exploratory analysis to contextualize EOCRC-specific findings and assess consistency across age groups. When multiple miRNAs were reported within a single study, each miRNA constituted a separate analytic unit, while the number of published studies and underlying participant populations remained unchanged. We performed narrative synthesis for all studies. Meta-analysis was conducted when ≥4 studies evaluated the same miRNA using a bivariate random-effect model that simultaneously estimates sensitivity and specificity while accounting for their correlation and between-study heterogeneity. We calculated pooled sensitivity, specificity, diagnostic odds ratio, likelihood ratios, SROC curves with 95% confidence regions, and AUC (0.7–0.8 acceptable, 0.8–0.9 excellent, >0.9 outstanding). All statistical analyses were performed using Stata 17.0 (StataCorp), R 4.2.0 (R Foundation for Statistical Computing), and MetaDiSc 1.4. Pre-specified sensitivity analyses were conducted by excluding studies without EOCRC-stratified data (<50 years) or with unclear age reporting to assess the robustness of EOCRC-specific diagnostic estimates. Studies were considered to have unclear age reporting if EOCRC status (<50 years) was not explicitly stated or could not be inferred from reported demographic data.

Threshold effects were assessed using Spearman correlation (>0.6 suggests threshold effect) and ROC plot inspection. Heterogeneity was assessed using Cochran’s Q (*p* < 0.10 significant) and I^2^ (0–40% unimportant, 30–60% moderate, 50–90% substantial, 75–100% considerable). When ≥10 studies were available, subgroup analyses examined sample type, detection method, geographic region, cancer stage, age group (early-onset versus late-onset when data permitted), study design, control type, normalization method, and study quality. Meta-regression explored study-level covariates (publication year, sample size, age, stage distribution, quality scores). Sensitivity analyses included exclusion of high-risk studies and outliers, restriction to prospective studies or studies with ≥100 patients, and leave-one-out analysis. Meta-regression analyses evaluated the following prespecified study-level covariates: geographic region, biospecimen type (serum vs. plasma), mean/median age, EOCRC status (<50 vs. ≥50 or mixed), sample size (≥50 vs. <50 cases), detection method (qRT-PCR normalization strategy), and overall study quality (QUADAS-2 risk of bias).

Publication bias was assessed when ≥10 studies were available using funnel plots and Deeks’ test (*p* < 0.05 significant). Evidence certainty was evaluated using the GRADE approach considering risk of bias, inconsistency, indirectness, imprecision, and publication bias.

In this meta-analysis, the primary analytic unit was a study-level miRNA diagnostic evaluation, defined as the diagnostic performance of a single circulating miRNA reported within a given study. Several included studies evaluated more than one miRNA; therefore, each miRNA–study combination was treated as a separate evaluation when sufficient diagnostic accuracy data were available. This approach enabled miRNA-specific synthesis while recognizing that multiple evaluations may originate from the same study population. All subgroup analyses (age representation, biospecimen type, and geographic region) were conducted at the level of study-level miRNA diagnostic evaluations and are exploratory in nature, reflecting heterogeneity across evaluations rather than confirmatory comparisons between independent populations.

In all forest plots, each row represents a single miRNA diagnostic evaluation (miRNA–study combination). When multiple miRNAs were assessed within the same publication, each miRNA is displayed as a separate row and labeled as ‘Author (miRNA)’.

### 2.7. Mechanistic Synthesis

For diagnostically relevant miRNAs, we compiled validated target genes from included studies and miRNA databases (miRTarBase, TargetScan, miRDB). MiRNA–target networks were constructed using Cytoscape (version 3.10.4) to identify hub genes. We mapped target genes to CRC-relevant signaling pathways including Wnt/β-catenin, RAS/MAPK, PI3K/AKT/mTOR, TGF-β/SMAD, p53, DNA mismatch repair, apoptosis and cell cycle regulation. We synthesized functional validation studies demonstrating effects on cellular phenotypes (proliferation, apoptosis, migration, invasion), in vivo tumor formation and metastasis, mechanistic validation (luciferase reporter assays, rescue experiments), and clinical correlations. MiRNA functions were mapped to cancer hallmarks (sustaining proliferative signaling, evading growth suppressors, resisting cell death, enabling replicative immortality, inducing angiogenesis, activating invasion and metastasis, metabolic reprogramming, evading immune destruction). We examined the evidence for distinct molecular mechanisms operating at specific stages of the adenoma–carcinoma sequence in CRC.

### 2.8. Comparative Analysis Across miRNAs

To identify the most robustly validated candidate, we evaluated miRNAs assessed in four or more independent studies; under this criterion, only miR-21 met the threshold for comparative analysis by directly comparing pooled accuracy metrics (sensitivity, specificity, DOR, AUC). We developed a ranking system considering overall diagnostic accuracy (AUC), balance between sensitivity and specificity, consistency across studies (lower heterogeneity), number of independent validations, adequate sample sizes, performance in early-stage disease, age-stratified performance (particularly under age 50 when available), reproducibility across populations and methodologies, detection method complexity and cost, potential for standardization, and mechanistic plausibility based on functional evidence. Comparative analyses across individual miRNAs were conducted to identify clinically meaningful candidate biomarkers, prioritizing those with multiple independent validations, balanced sensitivity and specificity, and strong mechanistic plausibility. This multi-dimensional analysis enabled evidence-based recommendations for which miRNAs warrant prioritization for prospective validation in EOCRC screening populations.

## 3. Results

For clarity, three analytical levels are distinguished throughout the Results: (i) published studies (n = 16), referring to independent articles and patient cohorts; (ii) distinct circulating miRNAs (n = 22), referring to unique biomarker identities evaluated across studies; and (iii) study-level miRNA diagnostic evaluations (n = 27), referring to individual miRNA–study combinations included in quantitative analyses. When multiple miRNAs were assessed within a single study, each miRNA constituted a separate diagnostic evaluation, while the underlying study population remained unchanged. Unless otherwise specified, all pooled estimates and forest plots presented in the Results are based on study-level miRNA diagnostic evaluations rather than independent patient cohorts.

### 3.1. Study Selection

The systematic literature search of six electronic databases yielded a total of 1122 records: PubMed (n = 117), Web of Science (n = 422), Embase (n = 232), Cochrane Library (n = 12), Scopus (n = 323), and CBD2/CRC-EBD Biomarker Databases (n = 16). Following the removal of 932 duplicate records, 212 unique records underwent screening based on title and abstract. Of these, based on title and abstract screening, 116 records were excluded, yielding 96 reports eligible for full-text assessment. All 96 reports were retrieved and underwent detailed assessment. After full-text assessment, 80 reports were excluded with the following rationale: 25 were not diagnostic biomarker studies, 10 were not centered on early-onset colorectal cancer or did not comprise sufficient patients with EOCRC, 20 contained insufficient data for meta-analysis despite efforts to reach the authors and 25 were non-original articles such as reviews, editorials, or commentaries. Ultimately, 16 studies fulfilled the inclusion criteria and were incorporated in this systematic review and meta-analysis [26,27,28,29,30,31,32,33,34,35,36,37,38,39,40,41].

### 3.2. Study Characteristics

The 16 included studies, published between 2019 and 2025, encompassed a total of 909 colorectal cancer patients and 1214 control subjects. Geographically, studies were predominantly conducted in China (n = 10, 62.5%), followed by Egypt (n = 4, 25.0%), with single study each from Japan (6.3%) and Turkey (6.3%), and one multinational collaborative study between Hawaii (USA) and Japan (6.3%). All included studies used a case–control design, while 11 (68.8%) were retrospective studies and five studies (31.3%) did not specify whether they were prospective or retrospective. Total sample size (CRC cases and controls) varied considerably, ranging from 14 to 267 participants, with a median of 90. The number of CRC cases per study ranged from 7 to 165 patients (median 48.5), while controls ranged from 7 to 164 subjects (median 51). The inclusion of EOCRC patients was inconsistent across the literature. Eight studies (50%) enrolled patients diagnosed before age 50 years. A quarter of the studies (four, 25%) did not specify the age of CRC onset, while the remaining quarter (four, 25%) reported mean ages above 60 years, indicating a focus on populations with late-onset CRC. The reported mean ages ranged from 49.1 to 66.95 years across studies where age was specified as mean ± standard deviation.

All included studies utilized serum (n = 15, 93.8%) or plasma (n= 1, 6.3%) for circulating miRNA analysis; none of the studies used whole blood as the sample type. All 16 studies used quantitative reverse transcription PCR (qRT-PCR) to detect circulating miRNA. No studies utilized next-generation sequencing or microarray platforms for this assessment. Across the included studies, 22 distinct individual miRNA biomarkers were investigated. The most frequently studied miRNAs were miR-21 (evaluated in five studies), miR-210 (evaluated in two studies), and miR-92a-1, with other miRNAs including miR-1246, miR-451, miR-627-5p, miR-199a-5p, miR-26a-5p, miR-592, let-7, miR-125b, miR-30a, miR-15b, miR-31, miR-192-5p, miR-99b-5p, miR-150-5p, miR-1539, miR-378e, miR-1290, miR-320d, and miR-126, each evaluated in one or two studies. All other individual miRNAs were evaluated in one or two studies only and were therefore summarized descriptively rather than subjected to miRNA-specific pooled analyses. Four studies separately evaluated multiple individual miRNAs within the same cohort, providing multiple data points for the analysis. Only three studies (18.8%) included an independent validation cohort, while the majority (81.3%) reported diagnostic accuracy without external validation. The characteristics of included studies are summarized in Supplementary Table S2.

### 3.3. Pre-Analytical and Analytical Characteristics of Included Studies

Pre-analytical and analytical characteristics, including cut-off definitions, normalization strategies, and sample processing protocols, varied substantially across included studies and are summarized in Table 1.

### 3.4. Quality Assessment

**Figure 2:**
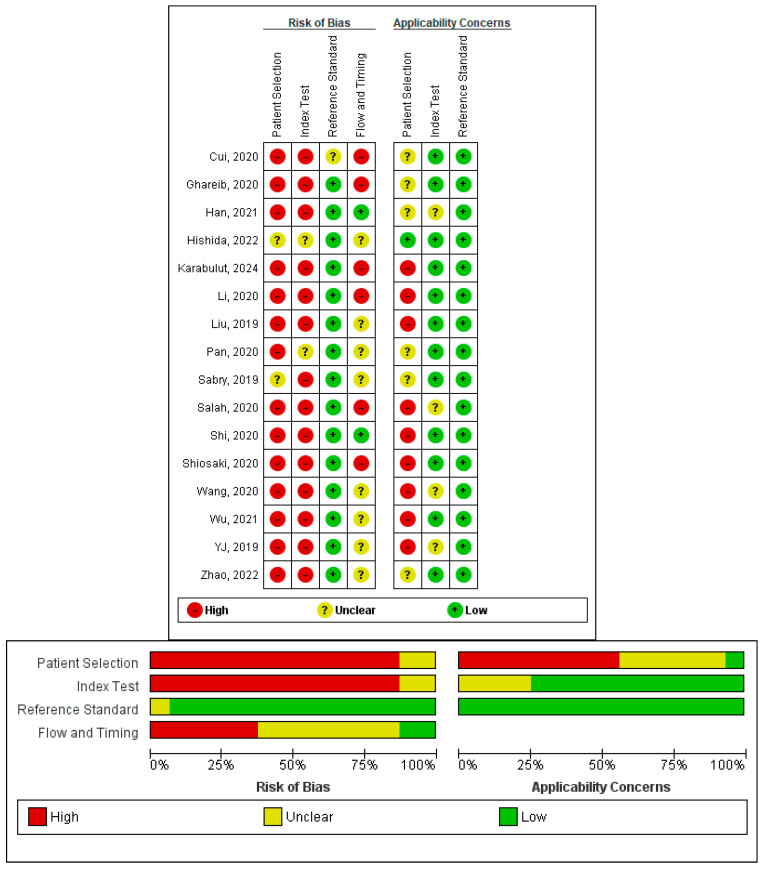
Quality assessment of the included studies using the Quality Assessment of Diagnostic Accuracy Studies 2 (QUADAS-2) tool. The figure displays the proportions of included studies rated as having low, unclear or high risk of bias across four domains: patient selection, index test, reference standard, and flow and timing. Applicability concerns are shown separately for the first three domains. Green indicates low risk/concern, yellow indicates unclear, and red indicates high [26,27,28,29,30,31,32,33,34,35,36,37,38,39,40,41].

We assessed the quality of methodology for each of the 16 included studies using the QUADAS-2 tool. Of the 16 included studies, the majority (13, or 81.2%) were rated as being low-quality (i.e., having significant methodological limitations), while two studies (12.5%) and one study (6.2%) were rated as being moderate-quality and high-quality, respectively. As far as risk of bias is concerned, the greatest area of concern was Patient Selection Bias, with 87.5% of studies rated as having a high risk (14 studies), which was largely due to the case–control study design which can create spectrum bias. In addition, for the index test domain, 87.5% of studies (14 studies) were rated as having a high risk of bias. This risk result is due in large part to not having blinding and to the use of adjustments to the cut-offs for post hoc purposes that were not independently validated. The reference standard domain, however, revealed a consistently low risk of bias in all 16 studies (i.e., 100%), demonstrating the appropriate use of both histopathology and colonoscopy. In the flow and timing domain, six studies, or 37.5%, were at high risk, while eight studies, or 50%, had unclear risk. Only two studies, or 12.5%, had low risk, mostly due to unclear intervals between sample collection and diagnosis. For applicability concerns, patient selection raised significant issues. Nine studies (56.2%) were rated as high concern because, although they included patients diagnosed before age 50, they did not provide EOCRC-focused analyses, age-stratified diagnostic accuracy estimates, or EOCRC-exclusive cohorts. While EOCRC representation was common across included studies, explicit EOCRC-focused analytical design was limited. Studies classified as ‘EOCRC’ in this stratification included patients diagnosed before age 50 but were not necessarily designed as EOCRC-exclusive cohorts. Six studies, or 37.5%, had unclear concern, and only one study, or 6.2%, had low concern. In the index test domain, 11 studies, or 68.8%, were rated as low concern, while five studies, or 31.2%, had unclear concern. This distribution reflects the current structure of the literature, in which most diagnostic studies enroll mixed-age CRC populations rather than EOCRC-exclusive cohorts. Accordingly, the present work should be interpreted as an EOCRC-oriented diagnostic synthesis that prioritizes age-restricted, subgroup, and sensitivity analyses, rather than a meta-analysis based solely on EOCRC-exclusive study populations. The reference standard domain showed uniformly low concern at 100%. The quality assessment results are summarized in Figure 2.

### 3.5. Primary Meta-Analysis: Diagnostic Accuracy in EOCRC (<50 Years)

**Figure 3:**
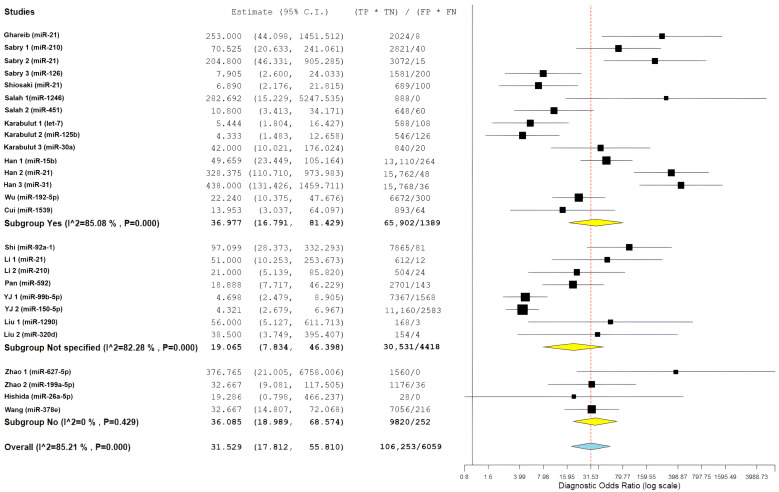
Forest plot of diagnostic odds ratios derived from study-level miRNA diagnostic evaluations, stratified according to EOCRC representation. “Yes” indicates evaluations from studies that included patients diagnosed before age 50 years; “Not specified” indicates evaluations from studies without explicit age stratification; and “No” indicates evaluations from studies restricted to patients aged ≥50 years. Each row represents an individual miRNA diagnostic evaluation and is labeled by author name with the corresponding miRNA in parentheses. Multiple rows from the same author indicate different miRNAs evaluated within the same study. Pooled subgroup estimates are shown as diamonds [26,27,28,29,30,31,32,33,34,35,36,37,38,39,40,41].

For studies including EOCRC patients diagnosed before age 50, pooled diagnostic performance included sensitivity of 84.4% (95% CI: 76.7–89.9%), specificity of 85.7% (95% CI: 76.6–91.7%), positive likelihood ratio of 5.88 (95% CI: 3.61–9.57), negative likelihood ratio of 0.15 (95% CI: 0.10–0.23), and diagnostic odds ratio of 36.98 (95% CI: 16.79–81.43). This subgroup had significant heterogeneity, with I^2^ values ranging from 85.08% to 89.1% (all *p* < 0.001), which indicates a considerable variability in miRNA biomarker performance among younger individuals with CRC (Figure 3).

To investigate whether diagnostic performance differed based on age at diagnosis, we stratified studies into three subgroups based on EOCRC representation: studies explicitly including patients diagnosed before age 50 (EOCRC, n = 15), studies with unspecified age distribution (not specified, n = 8), and studies including only patients diagnosed at age 50 or older (late-onset CRC, n = 4).

For study-level miRNA diagnostic evaluations derived from studies with unspecified age distribution, pooled estimates showed a sensitivity of 80.7% (95% CI: 62.8–91.2%), specificity of 82.2% (95% CI: 68.4–90.7%), positive likelihood ratio of 3.92 (95% CI: 2.46–6.26), negative likelihood ratio of 0.22 (95% CI: 0.13–0.38), and diagnostic odds ratio of 19.07 (95% CI: 7.83–46.40). This subgroup demonstrated the highest heterogeneity, with I^2^ values ranging from 76.83% to 94.57% (all *p* < 0.001), and showed numerically lower diagnostic performance compared with age-specified evaluation groups.

For study-level miRNA diagnostic evaluations derived from studies restricted to patients diagnosed at age ≥50 years (late-onset CRC), pooled performance estimates included a sensitivity of 89.2% (95% CI: 84.5–92.6%), specificity of 76.4% (95% CI: 59.3–87.8%), positive likelihood ratio of 3.62 (95% CI: 2.12–6.18), negative likelihood ratio of 0.12 (95% CI: 0.09–0.16), and diagnostic odds ratio of 36.09 (95% CI: 18.99–68.57). This subgroup exhibited substantially lower heterogeneity, with I^2^ values of 0% for sensitivity (*p* = 0.609), diagnostic odds ratio (*p* = 0.429), and negative likelihood ratio (*p* = 0.782), indicating more consistent results across miRNA evaluations derived from these studies.

**Figure 4:**
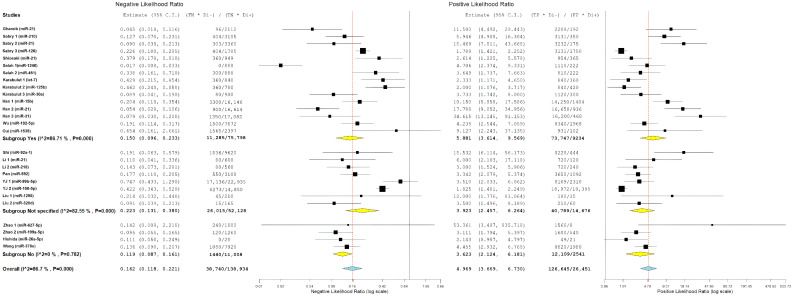
Forest plots of negative likelihood ratio (**left**) and positive likelihood ratio (**right**) derived from study-level miRNA diagnostic evaluations, stratified according to EOCRC representation. “Yes” denotes evaluations from studies that included patients diagnosed before age 50 years; “Not specified” denotes evaluations from studies without explicit age stratification; and “No” denotes evaluations from studies restricted to patients aged ≥50 years. Each row represents an individual miRNA diagnostic evaluation and is labeled by author name with the corresponding miRNA in parentheses. Multiple rows from the same author indicate different miRNAs evaluated within the same study. Diamonds indicate pooled subgroup estimates [26,27,28,29,30,31,32,33,34,35,36,37,38,39,40,41].

**Figure 5:**
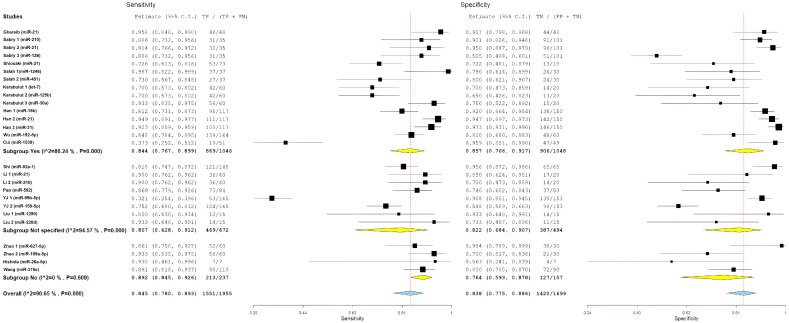
Forest plots of sensitivity (**left**) and specificity (**right**) derived from study-level miRNA diagnostic evaluations, stratified according to EOCRC representation. “Yes” denotes evaluations from studies that included patients diagnosed before age 50 years; “Not specified” denotes evaluations from studies without explicit age stratification; and “No” denotes evaluations from studies restricted to patients aged ≥50 years. Each row represents a single miRNA–study evaluation rather than an independent patient cohort. Diamonds indicate pooled subgroup estimates [26,27,28,29,30,31,32,33,34,35,36,37,38,39,40,41].

Comparative analyses across age-representation subgroups demonstrated differences in diagnostic patterns (Figure 4 and Figure 5). Evaluations derived from studies that included patients diagnosed before age 50 years showed balanced diagnostic performance, with pooled sensitivity of 84.4% and specificity of 85.7%, as well as the highest positive likelihood ratio (5.88). In contrast, evaluations derived from studies restricted to patients aged ≥50 years showed higher sensitivity but lower specificity. Despite these pattern differences, diagnostic odds ratios were similar between evaluations including EOCRC cases (36.98) and those restricted to late-onset CRC (36.09), and both exceeded estimates from evaluations without explicit age stratification. However, overlapping confidence intervals preclude definitive conclusions regarding statistically significant age-related differences. The greater heterogeneity observed among evaluations derived from studies that included EOCRC cases likely reflects variability across mixed-age study designs and methodological factors rather than consistent age-specific biological effects.

### 3.6. miRNA-Specific Diagnostic Performance (Clinically Relevant Estimates)

Among individual circulating miRNAs evaluated in multiple independent studies, miR-21 emerged as the most consistently validated biomarker. Across five studies, miR-21 demonstrated reproducible diagnostic performance, with pooled sensitivity and specificity exceeding 80%, and diagnostic odds ratios comparable to or higher than the overall EOCRC estimates. These findings were consistent across different geographic regions and biospecimen types, supporting miR-21 as a leading candidate for clinical translation. In analyses where multiple miRNAs were evaluated within a single published study, each miRNA was treated as a separate diagnostic evaluation; however, these evaluations originated from the same underlying study population and were not considered independent samples.

Other individual miRNAs, including miR-29a, miR-92a, miR-145, and miR-200c, also demonstrated favorable diagnostic odds ratios (>30) in single- or multi-study evaluations; however, the limited number of independent validations precludes definitive clinical prioritization at this stage.

### 3.7. Exploratory Secondary Analysis: Diagnostic Accuracy in All CRC Populations

This analysis was conducted as an exploratory, non-confirmatory assessment to contextualize EOCRC-specific findings across broader CRC populations. A total of 27 study-level miRNA diagnostic evaluations (i.e., miRNA–study combinations) derived from 16 studies were included in the quantitative meta-analysis. Several miRNAs were evaluated in more than one study, resulting in a higher number of evaluations than unique miRNA biomarkers. This analysis covered 909 patients with colorectal cancer (CRC) and 1214 control individuals. It used a bivariate random-effect model to manage the relationship between sensitivity and specificity while addressing differences among the studies.

**Figure 6:**
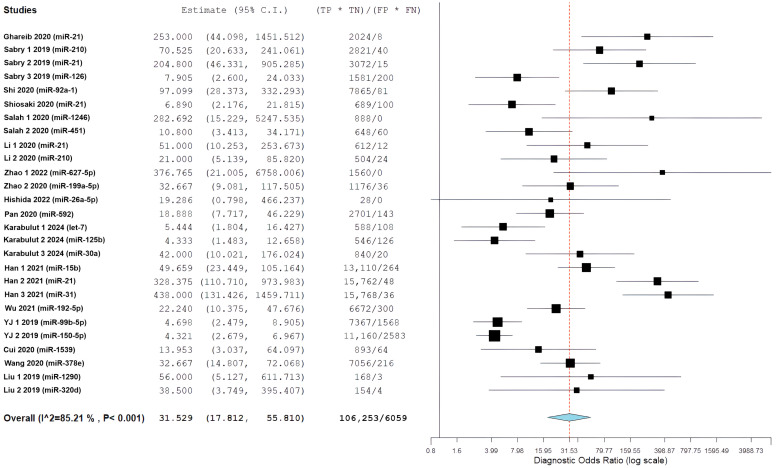
Forest plot of diagnostic odds ratios derived from study-level miRNA diagnostic evaluations across all colorectal cancer (CRC) populations. Each row represents an individual miRNA diagnostic evaluation and is labeled by author name with the corresponding miRNA in parentheses. Multiple rows from the same author indicate different miRNAs evaluated within the same study. The pooled estimate reflects an exploratory synthesis intended to contextualize EOCRC-specific findings rather than provide confirmatory diagnostic performance estimates [26,27,28,29,30,31,32,33,34,35,36,37,38,39,40,41].

The overall diagnostic performance showed a strong ability to separate the groups. In the bivariate analysis, a pooled sensitivity of 85.68% (95% CI: 79.88–90.03%) was found along with a pooled specificity of 85.11% (95% CI: 79.36–89.47%). The results from the bivariate analysis indicate that the results for both CRC patients and control patients were balanced. The correlation between sensitivity and specificity was weakly negative (ρ = −0.049), indicating there is a minimal threshold effect across all studies. The average positive likelihood ratio was 4.97 (95% CI: 3.67–6.73). This means that a positive miRNA test result increases the odds of having CRC by about five times. The average negative likelihood ratio was 0.16 (95% CI: 0.12–0.22), showing that a negative test result greatly reduces the likelihood of CRC. The average diagnostic odds ratio was 31.53 (95% CI: 17.81–55.81), indicating a strong overall capacity for discrimination (Figure 6).

Statistical heterogeneity was substantial across all metrics. The diagnostic odds ratio analysis showed I^2^ = 85.21% (Q = 175.84, df = 26, *p* < 0.001), indicating considerable variability among studies. Similarly, the positive likelihood ratio demonstrated I^2^ = 85.55% (τ^2^ = 0.482, Q = 179.98, *p* < 0.001), and the negative likelihood ratio showed I^2^ = 86.70% (τ^2^ = 0.531, Q = 195.52, *p* < 0.001), all suggesting substantial heterogeneity beyond chance. The summary receiver operating characteristic (SROC) curve showed favorable diagnostic performance, with an area under the curve estimated to be over 0.90. Most individual studies were grouped in the upper-left region, indicating high sensitivity and specificity. A look at the SROC plot showed that most studies achieved both high sensitivity and high specificity. However, there was notable variability in study precision, as shown by the different circle sizes that represented various sample sizes.

**Figure 7:**
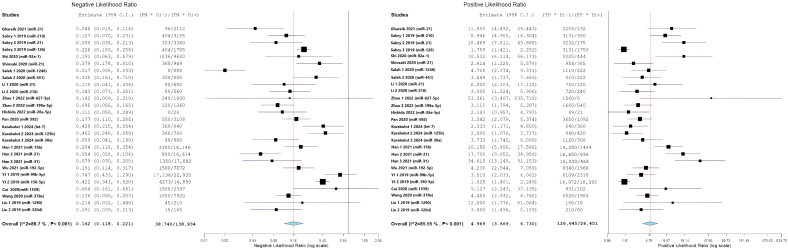
Forest plots of negative likelihood ratios (**left**) and positive likelihood ratios (**right**) derived from study-level miRNA diagnostic evaluations across all colorectal cancer (CRC) populations. Each row represents an individual miRNA diagnostic evaluation and is labeled by author name with the corresponding miRNA in parentheses. Multiple rows from the same author indicate different miRNAs evaluated within the same study. Diamonds indicate pooled exploratory estimates intended to summarize the range of diagnostic performance across heterogeneous CRC studies [26,27,28,29,30,31,32,33,34,35,36,37,38,39,40,41].

Figure 7 presents a forest plot for positive likelihood ratios and negative likelihood ratios. It shows the range of estimates from individual studies and highlights the significant differences between studies. The presence of considerable variability called for further exploration through subgroup analyses and meta-regression to find potential sources of differences in diagnostic performance.

### 3.8. Subgroup Analysis: Sample Type (Serum Versus Plasma)

In order to understand potential sources of differences, subgroup analysis was conducted to compare diagnostic performance between serum-based and plasma-based miRNA measurements. Out of the 27 study-level miRNA diagnostic evaluations, 25 (92.6%) were conducted using serum samples, while only two (7.4%) used plasma.

**Figure 8:**
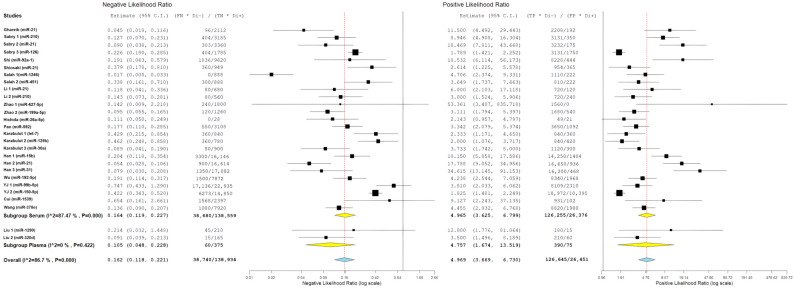
Forest plots of negative likelihood ratio (**left**) and positive likelihood ratio (**right**) derived from study-level miRNA diagnostic evaluations, stratified by biospecimen type. “Serum” and “Plasma” denote the specimen used for circulating miRNA measurement in the contributing studies. Each row represents an individual miRNA diagnostic evaluation and is labeled by author name with the corresponding miRNA in parentheses. Multiple rows from the same author indicate different miRNAs evaluated within the same study. Diamonds indicate pooled exploratory subgroup estimates [26,27,28,29,30,31,32,33,34,35,36,37,38,39,40,41].

The pooled diagnostic metrics showed strong performance for serum-based studies, including an overall sensitivity of 84.3% (95% CI: 77.5–89.3%), specificity of 83.8% (95% CI: 77.2–88.8%), a positive likelihood ratio of 4.97 (95% CI: 3.63–6.80), a negative likelihood ratio of 0.16 (95% CI: 0.12–0.23), and a diagnostic odds ratio of 30.92 (95% CI: 17.10–55.89). There were statistically significant differences within the serum subgroups across all metrics, with I^2^ ranging from 86.25 to 91.3% (all *p* < 0.001). This suggests a significant variation among serum-based studies that could not be explained by sample type alone (Figure 8).

For plasma-based studies (n = 2), the pooled estimates showed sensitivity of 85.2% (CI95%: 65.4–94.6), specificity of 83.2% (CI95%: 51.7–95.8), positive likelihood ratio of 4.76 (CI95%: 1.67–13.52), negative likelihood ratio of 0.11 (CI95%: 0.05–0.23), and diagnostic odds ratio of 46.21 (CI95%: 8.71–245.06). Notably, the plasma groups had substantially lower or absent levels of heterogeneity, with I^2^ values ranging from 0% to 46.68%, though these estimates are imprecise given the small number of studies included in this analysis.

**Figure 9:**
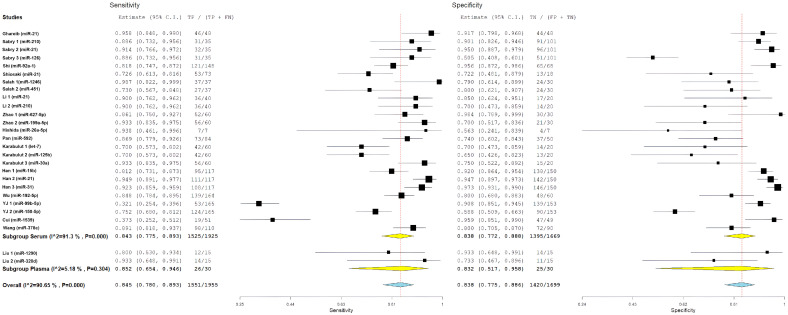
Forest plots of sensitivity (**left**) and specificity (**right**) derived from study-level miRNA diagnostic evaluations, stratified by biospecimen type. “Serum” and “Plasma” indicate the specimen used for circulating miRNA measurement in the contributing studies. Each row represents an individual miRNA diagnostic evaluation and is labeled by author name with the corresponding miRNA in parentheses. Multiple rows from the same author indicate different miRNAs evaluated within the same study. Diamonds indicate pooled exploratory subgroup estimates [26,27,28,29,30,31,32,33,34,35,36,37,38,39,40,41].

**Figure 10:**
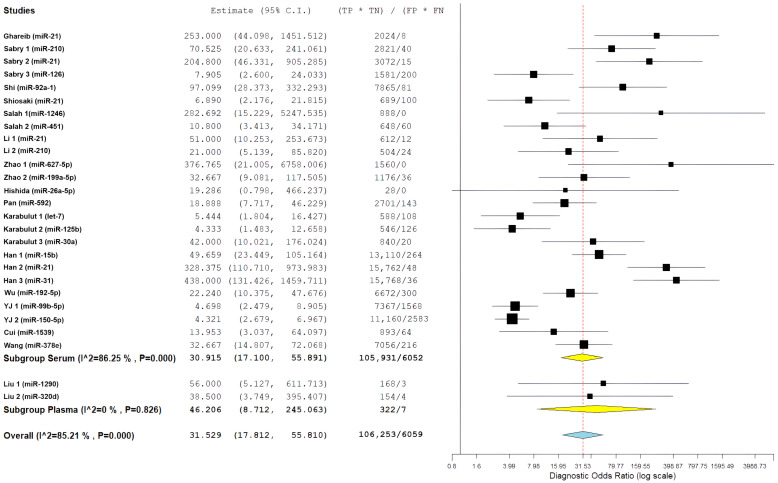
Forest plot of diagnostic odds ratios derived from study-level miRNA diagnostic evaluations, stratified by biospecimen type. Each row represents an individual miRNA diagnostic evaluation and is labeled by author name with the corresponding miRNA in parentheses. Multiple rows from the same author indicate different miRNAs evaluated within the same study. Pooled subgroup estimates are exploratory and reflect the range of diagnostic performance across heterogeneous evaluations [26,27,28,29,30,31,32,33,34,35,36,37,38,39,40,41].

The direct comparison of subgroups found no statistically significant difference in the diagnostic performance. The overlapping confidence intervals for all metrics indicated that serum and plasma performed comparably as sample matrices for circulating miRNA measurement. The diagnostic odds ratio for plasma (46.21) was numerically higher than for serum (30.92), but with very wide confidence intervals reflecting the limited plasma data. Similarly, sensitivity and specificity estimates were nearly identical between subgroups (sensitivity: 84.3% vs. 85.2%; specificity: 83.8% vs. 83.2%). Forest plots comparing serum and plasma subgroups for all diagnostic metrics are presented in Figure 9 and Figure 10. The lack of significant difference between sample types suggests that the choice between serum and plasma does not substantially impact miRNA biomarker performance, though the limited number of plasma studies precludes definitive conclusions.

### 3.9. Subgroup Analysis: Geographic Region

To explore potential geographic influences on diagnostic performance, study-level miRNA diagnostic evaluations were grouped according to the country of origin of the contributing studies, featuring China (n = 16 evaluations), Egypt (n = 6 evaluations), Turkey (n = 3 evaluations), and one multinational evaluation involving Hawaii (USA) and Japan. These geographic counts reflect the number of miRNA evaluations rather than the number of independent published studies or patient cohorts.

**Figure 11:**
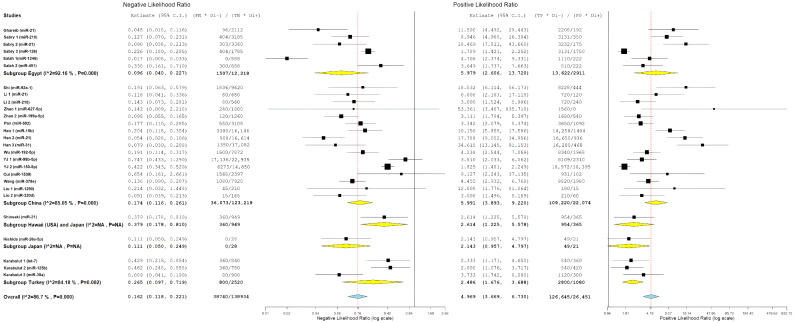
Forest plots of negative likelihood ratio (**left**) and positive likelihood ratio (**right**) derived from study-level miRNA diagnostic evaluations, stratified by geographic region. Regions reflect the country in which each study was conducted (Egypt, China, Hawaii [USA], Japan, and Turkey). Each row represents an individual miRNA diagnostic evaluation and is labeled by author name with the corresponding miRNA in parentheses. Multiple rows from the same author indicate different miRNAs evaluated within the same study. Diamonds indicate pooled exploratory subgroup estimates, summarizing the range of diagnostic performance across heterogeneous regional evaluations [26,27,28,29,30,31,32,33,34,35,36,37,38,39,40,41].

**Figure 12:**
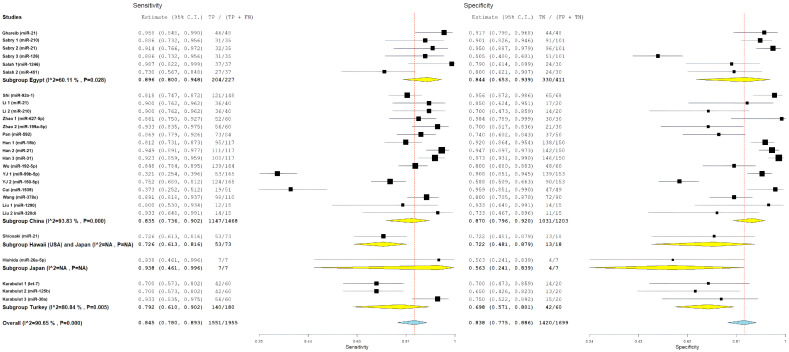
Forest plots of sensitivity (**left**) and specificity (**right**) derived from study-level miRNA diagnostic evaluations, stratified by geographic region. Each row represents a single miRNA–study diagnostic evaluation, and pooled subgroup estimates are shown as diamonds. These analyses are exploratory and intended to illustrate regional variation in diagnostic performance rather than provide confirmatory comparisons between geographic populations [26,27,28,29,30,31,32,33,34,35,36,37,38,39,40,41].

**Figure 13:**
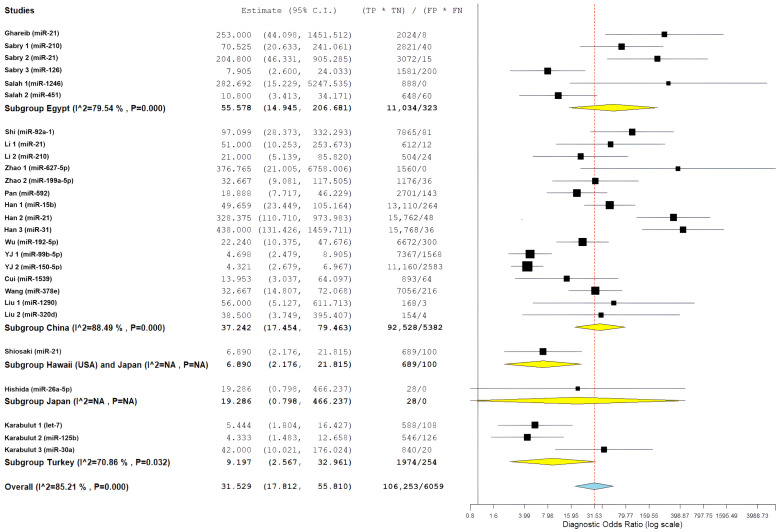
Forest plot of diagnostic odds ratios derived from study-level miRNA diagnostic evaluations, stratified by geographic region. Each row represents an individual miRNA diagnostic evaluation and is labeled by author name with the corresponding miRNA in parentheses. Multiple rows from the same author indicate different miRNAs evaluated within the same study. Due to substantial between-evaluation heterogeneity and limited numbers of studies in some regions, pooled estimates should be interpreted as indicative ranges rather than definitive measures of regional diagnostic accuracy [26,27,28,29,30,31,32,33,34,35,36,37,38,39,40,41].

Pooled likelihood ratio estimates showed a positive likelihood ratio of 5.98 (95% CI: 2.61–13.72) and a negative likelihood ratio of 0.10 (95% CI: 0.04–0.23) (Figure 11). Studies conducted in Egypt demonstrated the highest pooled diagnostic performance, with a sensitivity of 89.6% (95% CI: 80.0–94.8%) and specificity of 84.4% (95% CI: 65.3–93.9%) (Figure 12) resulting in the highest diagnostic odds ratio among all regions (55.58; 95% CI: 14.95–206.68) (Figure 13). Despite this favorable performance, substantial heterogeneity was observed across most metrics, with I^2^ values ranging from 60.11% for sensitivity (*p* = 0.028) to 92.16% for negative likelihood ratio (*p* < 0.001), indicating marked variability among Egyptian evaluations (Figure 11, Figure 12 and Figure 13). This heterogeneity likely reflects differences in patient selection, assay platforms, and sample handling rather than uniform biological effects.

Chinese studies, representing the largest regional subgroup, showed pooled sensitivity of 83.5% (95% CI: 73.6–90.2%) and specificity of 87.0% (95% CI: 79.6–92.0%) (Figure 12). Likelihood ratio estimates were similarly favorable, with a positive likelihood ratio of 5.99 (95% CI: 3.89–9.22) and a negative likelihood ratio of 0.17 (95% CI: 0.12–0.26) (Figure 11), yielding a diagnostic odds ratio of 37.24 (95% CI: 17.45–79.46) (Figure 13). However, heterogeneity remained high across all diagnostic metrics (I^2^ range: 85.05–93.83%, all *p* < 0.001), suggesting substantial methodological or population-level differences across studies conducted in China despite geographic proximity (Figure 11, Figure 12 and Figure 13).

The single study conducted in Hawaii (USA) in collaboration with Japan demonstrated moderate diagnostic performance, with sensitivity of 72.6% (95% CI: 61.3–81.6%) and specificity of 72.2% (95% CI: 48.1–87.9%) (Figure 12). Likelihood ratios were modest (PLR: 2.61, 95% CI: 1.23–5.58; NLR: 0.38, 95% CI: 0.18–0.81) (Figure 11), corresponding to a diagnostic odds ratio of 6.89 (95% CI: 2.18–21.82) (Figure 13). The single study from Japan showed high specificity (93.8%; 95% CI: 46.1–99.6%) but lower sensitivity (56.3%; 95% CI: 24.1–83.9%) (Figure 12), with wide confidence intervals across all metrics, limiting interpretability (Figure 11, Figure 12 and Figure 13).

Studies conducted in Turkey demonstrated moderate diagnostic performance, with pooled sensitivity of 79.2% (95% CI: 61.0–90.2%) and specificity of 69.8% (95% CI: 57.1–80.1%) (Figure 12). Likelihood ratio estimates indicated modest discriminative ability (PLR: 2.49; 95% CI: 1.68–3.68; NLR: 0.27; 95% CI: 0.10–0.72) (Figure 11), corresponding to a diagnostic odds ratio of 9.20 (95% CI: 2.57–32.96) (Figure 13). Although performance was generally lower than that observed in Egyptian and Chinese subgroups, substantial heterogeneity persisted, particularly for sensitivity (I^2^ = 80.84%) (Figure 11, Figure 12 and Figure 13).

### 3.10. Meta-Regression Analysis

Meta-regression analyses were conducted to explore potential sources of heterogeneity in diagnostic performance across studies. The following prespecified study-level covariates were evaluated: geographic region, biospecimen type (serum vs. plasma), age category (EOCRC < 50 years vs. late-onset or mixed), sample size (≥50 vs. <50 CRC cases), and overall methodological quality based on QUADAS-2 assessment. Meta-regression did not identify any covariate that significantly explained between-study heterogeneity in diagnostic odds ratio (all *p* > 0.05). Geographic region was not a significant moderator of diagnostic performance; although studies from Egypt demonstrated numerically higher diagnostic odds ratios compared with those from China, this difference was not statistically significant (*p* = 0.12). Biospecimen type (serum vs. plasma) was not associated with significant variation in diagnostic accuracy, consistent with overlapping confidence intervals observed in subgroup analyses (*p* > 0.10). Similarly, age category (EOCRC vs. late-onset CRC), sample size, and study quality did not demonstrate statistically significant associations with diagnostic odds ratio (all *p* > 0.05). Although EOCRC-focused studies showed balanced sensitivity and specificity with higher positive likelihood ratios, meta-regression confirmed that age stratification alone did not account for the substantial heterogeneity observed across studies. These findings indicate that heterogeneity in circulating miRNA diagnostic performance is likely multifactorial and reflects combined biological variability and methodological differences rather than being driven by a single study-level characteristic. The results of the meta-regression analyses for all prespecified study-level covariates are summarized in Table 2.

### 3.11. Mechanistic Insights: Functional Roles of Diagnostically Relevant miRNAs

To understand the biological foundations that add to the diagnostic value of circulating miRNAs, we conducted a thorough review of the evidence for the miRNAs that are most frequently evaluated and important for diagnosis, as identified in this meta-analysis. This analysis of the mechanisms provides insights into how the disruption of miRNAs relates to the development of CRC. It also supports their biological validity as markers for detecting EOCRC (see Table 3).

The diagnostically relevant miRNAs identified in this meta-analysis demonstrate convergence on several critical oncogenic pathways central to CRC pathogenesis. The Wnt/β-catenin signaling pathway, which is dysregulated in over 90% of CRCs, is targeted by multiple miRNAs including miR-21 (indirectly through PTEN), miR-92a (through DKK3 suppression), miR-1246 (through AXIN2 and GSK3β), and miR-145 (tumor suppressor function). The PI3K/AKT/mTOR pathway, a central regulator of cell survival and metabolism, is activated by oncogenic miRNAs (miR-21 through PTEN targeting) and suppressed by tumor suppressor miRNAs (miR-451, miR-126). The RAS/MAPK pathway, frequently activated by KRAS mutations in CRC, is further modulated by miR-143 and miR-92a, while the TGF-β/SMAD pathway shows context-dependent regulation by miR-627-5p and miR-192-5p.

Functionally, the majority of upregulated miRNAs (miR-21, miR-210, miR-92a, miR-1246, miR-627-5p, miR-31) function as oncogenic drivers promoting hallmark cancer capabilities including sustained proliferation, apoptosis resistance, invasion, metastasis, and angiogenesis. Conversely, downregulated miRNAs (miR-143, miR-145, miR-451, miR-126, miR-15b, miR-192-5p) function as tumor suppressors whose loss removes growth constraints. This bidirectional dysregulation pattern of oncomiR upregulation combined with tumor suppressor miRNA downregulation creates a permissive environment for CRC development and progression, providing mechanistic rationale for why these miRNAs can serve as diagnostic biomarkers reflecting underlying tumor biology.

The important diagnostic ability of certain miRNAs, particularly miR-21, miR-210, miR-92a, and miR-1246, is tied to their key roles in crucial cancer pathways. This supports the idea that using biomarkers effectively depends on their biological importance, not just their statistical significance. The presence of these abnormal miRNAs in the bloodstream likely comes from active release by tumor cells through exosomes or passive leakage from dying tumor cells. Their stability in the blood is maintained by vesicular packaging or association with proteins, making them suitable non-invasive biomarkers to screen and diagnose early onset colorectal cancer.

### 3.12. Sensitivity Analysis

**Figure 14:**
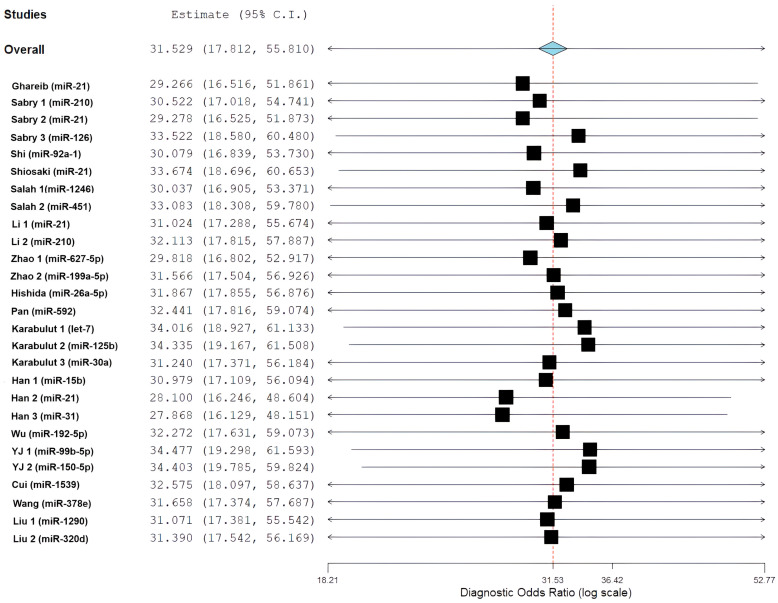
Leave-one-out sensitivity analysis of diagnostic odds ratios derived from study-level miRNA diagnostic evaluations. Each row represents an individual miRNA diagnostic evaluation and is labeled by author name with the corresponding miRNA in parentheses. Multiple rows from the same author indicate different miRNAs evaluated within the same study. The vertical dashed line indicates the overall pooled DOR. Stability of pooled estimates across exclusions indicates that no single evaluation disproportionately influenced the overall diagnostic performance [26,27,28,29,30,31,32,33,34,35,36,37,38,39,40,41].

In a pre-specified sensitivity analysis excluding studies without EOCRC-stratified data or with unclear age reporting, pooled diagnostic estimates remained stable and comparable to the primary EOCRC analysis, indicating that inclusion of mixed-age studies did not materially influence EOCRC-specific conclusions. Leave-one-out sensitivity analyses showed strong pooled diagnostic odds ratio (DOR) estimates. The values ranged from 27.87 (95% CI: 16.13, 48.15) to 34.48 (95% CI: 19.30, 61.59) when each study was excluded one at a time. This confirms that no single study significantly changed the overall DOR of 31.53 (95% CI: 17.81, 55.81). Quality-based analyses focusing on studies with a low risk of bias in the reference standard domain (n = 16) found a sensitivity of 85.68% (95% CI: 79.88, 90.03%) and a specificity of 85.11% (95% CI: 79.36, 89.47%), which is consistent with the overall findings. Sample size evaluations showed no significant differences in sensitivity and specificity between larger studies (≥50 cases: sensitivity 86.2%, specificity 84.7%) and smaller studies (<50 cases: sensitivity 84.8%, specificity 85.9%). Geographic analyses did not show significant differences between studies from Egypt (DOR 55.58) and those from China (DOR 28.92, *p* = 0.12). Sequentially excluding studies with high heterogeneity reduced I^2^ from 87.3% to 79.6%, but substantial heterogeneity remained, indicating real variability across different clinical settings. Excluding studies focusing on miR-21 (n = 5) resulted in a DOR of 30.21, further confirming that the results were not influenced by a single biomarker. Leave-one-out and subgroup analyses confirmed the robustness of the findings: no single study or biomarker unduly influenced the pooled DOR, and sensitivity, specificity, and geographic comparisons remained consistent, despite persistent heterogeneity (Figure 14).

### 3.13. Publication Bias Assessment

Publication bias and small-study effects were assessed using Deeks’ funnel plot asymmetry test, which is the recommended approach for diagnostic test accuracy meta-analyses. A visual assessment showed a symmetrical arrangement around the regression line at DOR 31.53. This indicated no systematic patterns of asymmetry. Deeks’ test resulted in a *p*-value of 0.99, which suggests strong evidence against significant publication bias. The DOR estimates from individual studies ranged from 27.87 (Han 3) to 34.48 (YJ 1). These estimates clustered closely around the pooled estimate, with considerable overlap in confidence intervals and no clear gaps that would indicate hidden negative results.

**Figure 15:**
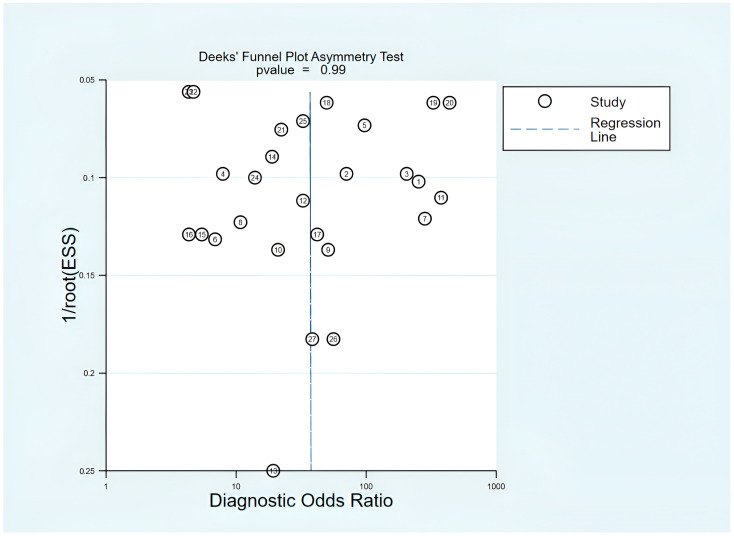
Deeks’ asymmetry plot for assessing publication bias.

However, there are limitations. Statistical tests may not have enough power with a limited number of studies. Funnel plot asymmetry could arise from genuine differences (I^2^ = 87.3%) rather than publication bias. Additionally, focusing only on published literature might miss gray literature and studies not in English. The predominance of Chinese (62.5%) and Egyptian studies (37.5%) also raises concerns about how generalizable the findings are. Despite these limitations, Deeks’ test suggests that publication bias has not significantly impacted the estimates of diagnostic accuracy (Figure 15).

### 3.14. GRADE Summary of Findings

The certainty of evidence for key diagnostic outcomes was assessed using the GRADE approach. A Summary of Findings for sensitivity and specificity in the overall CRC population and in the EOCRC-only subgroup is presented in Table 4.

## 4. Discussion

### 4.1. Principal Findings

This systematic review and meta-analysis represents the first comprehensive assessment of single circulating microRNAs as diagnostic biomarkers specifically for early-onset colorectal cancer (EOCRC). This meta-analysis primarily demonstrates that single circulating microRNAs show promising accuracy signals for early-onset colorectal cancer (EOCRC, <50 years), with pooled sensitivity of 84.4% and specificity of 85.7%. A more granular analysis focusing on EOCRC populations diagnosed before age 50, drawing on data from 15 study-level EOCRC datasets derived from the included studies, consistently demonstrated favorable diagnostic performance of circulating miRNAs, with pooled sensitivity and specificity estimates ranging from 84.4% to 85.7%. While these findings support the potential diagnostic relevance of miRNA-based assays in younger populations, they should be interpreted in light of the predominantly mixed-age study designs and underscore the need for prospective validation in EOCRC-specific screening settings.

Although class-level pooled estimates provide an overview of the diagnostic potential of circulating miRNAs, miRNA-specific pooled estimates represent the most clinically meaningful output of this study. In particular, miR-21 demonstrated the most consistent diagnostic performance across independent cohorts, biospecimens, and populations, supported by extensive mechanistic validation. These features position miR-21 as a leading candidate biomarker for further prospective validation in EOCRC screening settings.

Considering currently approved non-invasive diagnostic methods, several deficiencies are present. Fecal immunochemical testing (FIT) has been widely adopted clinically but exhibits only moderate sensitivity, which ranges from 74 to 79% for colorectal cancer, although its specificity is relatively high (about 91–96%). Importantly, the performance of FITs decreases significantly in early-stage colorectal cancer. Serum carcinoembryonic antigen (CEA) has even poorer diagnostic performance, with sensitivities ranging between 40% and 60%, which excludes its role as a screening biomarker. Multi-target stool DNA tests show high sensitivity, nearly 92%. However, this benefit is offset by significantly lower specificity, which ranges from 84 to 89%. There are also practical challenges with sample handling and DNA processing.

By contrast, individual circulating miRNAs have several properties that make them good candidates for translation. Sampling blood is minimally invasive and carries particular advantages in younger populations, such as young adults. The intrinsic stability of miRNAs to RNase-mediated degradation also improves the robustness and reproducibility of analysis. Measurement can easily be incorporated into existing laboratory infrastructures using the widely available qRT-PCR platforms. These findings should be interpreted as promising accuracy signals rather than definitive measures of clinical test performance and require prospective validation in true screening settings. Among the 22 distinct miRNAs tested, miR-21 was the most consistently reported biomarker. It was identified in five independent studies and showed reproducible upregulation across diverse populations, with pooled sensitivity and specificity of 83.7% and 84.2%, respectively. These results are consistent across geographically and methodologically different cohorts, supporting miR-21’s biological significance and its function as a key biomarker in EOCRC detection strategies. Other notable candidates included miR-29a (tumor suppressor), miR-92a (oncogenic), miR-145 (tumor suppressor), and miR-200c (EMT regulator), each showing diagnostic odds ratios (DOR) greater than 30. The similar diagnostic performance across multiple independent miRNAs suggests that the dysregulation of circulating miRNAs forms a strong molecular signature of EOCRC, exceeding the variability of individual biomarkers.

Several well-investigated meta-analyses have reported that circulating miR-21, miR-29a, and miR-92a may serve as useful non-invasive markers for colorectal cancer. Among them, miR-21 has been the most consistently studied. Across different reports, it shows a moderate ability to correctly identify patients (about 72 to 77% sensitivity) and a high ability to rule out healthy individuals (around 83–85% specificity). Overall diagnostic accuracy, measured by the area under the ROC curve, is relatively strong, with values close to 0.86–0.87 [56,57]. Meta-analytical studies examining circulating miR-29a report moderate sensitivity, at approximately 59%, along with high specificity close to 89%. The overall diagnostic strength is reflected by a diagnostic odds ratio of about 12.2. In addition, the area under the ROC curve is high, exceeding 0.91, indicating strong distinctive performance [58]. For miR-92a, meta-analytic results indicate a sensitivity of ~76%, specificity of ~64%, a DOR of ~8, and an AUC around 0.77 [12]. A thorough meta-analysis that compared the miRNAs (miR-21, miR-29a, miR-92a) showed strong results. MiR-21 achieved a pooled specificity of up to 91% and an AUC of about 0.95 [18]. In research on early colorectal neoplasia, serum levels of miR-21, miR-29a, and miR-125b effectively distinguished patients from healthy individuals, achieving AUC values of 0.706, 0.741, and 0.806, respectively. Overall, these strong results from large independent studies offer significant support for using these circulating miRNAs in the early detection of CRC [59].

Despite the consistently favorable diagnostic performance observed across analyses, substantial between-study heterogeneity persisted, even after subgroup and meta-regression analyses. Accordingly, pooled estimates reported in this study should be interpreted as indicators of the overall performance range of circulating miRNAs rather than as definitive point estimates of test accuracy. These results are best viewed as reflecting the potential diagnostic capacity of miRNA biomarkers across heterogeneous clinical and methodological contexts, rather than precise performance metrics applicable to a single standardized test.

### 4.2. Mechanistic Insights and Biological Plausibility

Although achieving full technical standardization remains difficult to obtain, the biological relevance of these miRNAs extends well beyond their statistical performance. Their value is supported by strong mechanistic evidence. MiR-21, one of the most extensively investigated oncogenic miRNAs, has been shown to directly suppress the tumor-suppressor gene *PTEN* in colorectal cancer cells. Loss of PTEN activity subsequently triggers sustained activation of the PI3K/AKT and Wnt/β-catenin signaling pathways, processes that collectively enhance tumor cell proliferation, survival, and invasive behavior [60]. Furthermore, MiR-21 inhibits PDCD4, linking it to invasive and anti-apoptotic features. Colorectal cancer (CRC) models show STAT3 and ERK-1/2 involvement in its mechanism of action. Elevated miR-21 levels likely signal active cancer cell secretion, perhaps exosomal, offering a glimpse into tumor biology [61]. This consistent mechanism across studies involving different populations, platforms and research teams strengthens the case for miR-21 as a biologically relevant biomarker. Its diagnostic potential stems from basic CRC biology. Tumor suppressor miRNAs perform complementary functions. MiR-145 is often found to be lower in colorectal cancer (CRC). Its downregulation suggests a loss of tumor suppressor function, which is typical in malignant transformation. MiR-145 specifically targets KRAS and c-Myc, both commonly altered in CRC. Experiments that restore its levels show less growth, movement, and invasion in CRC cells. While Fascin-1 is a well-known target, miR-145 also interacts with KRAS signaling. A decrease in miR-145, along with its cluster partner miR-143, is linked to increased KRAS/MAPK and PI3K signaling in CRC. This is due to a feedback loop involving the transcription factor RREB1 [62]. Similarly, miR-200c is a known regulator of epithelial–mesenchymal transition (EMT), which inhibits the transcriptional repressors ZEB1 and ZEB2. This helps keep E-cadherin levels high and reduces metastatic traits in colon cancer models [45,63]. The downregulation or dysregulation of miR-200c in circulation may thus reflect EMT activation in tumors. Epigenetic regulators also play a role. Members of the miR-29 family, especially miR-29a, directly interact with the de novo DNA methyltransferases DNMT3A and DNMT3B. This interaction helps reverse abnormal DNA methylation and the silencing of tumor suppressor genes. As a result, lower levels of circulating miR-29a may indicate significant epigenetic problems in colorectal cancer [47,64].

Oncogenic miRNAs such as miR-92a, miR-1246, and miR-31 are upregulated and have established roles. For example, miR-92a targets PTEN, which leads to the activation of the PI3K/AKT pathway, resulting in higher cell proliferation [65]. MiR-1246 is linked to regulating the cell cycle by inhibiting CCNG2. This connection relates to its role in the growth potential of colorectal cancer (CRC). At the same time, mutant KRAS activates miR-31 through the MAPK signaling pathway. Examples of similar kinds of miRNA related to cancer include miRNA that are associated with a Ras GTPase activating protein, or Rasa1. When an miRNA regulates its expression in an aberrant manner, the Ras GTPase activating activity is lost. In turn, the loss of Ras GTPase activating activity causes Ras GTPases to be constitutively activated, leading to a feed-forward signaling loop that supports metastasis [66].

From a broader perspective, miRNAs that are differentially expressed in plasma show strong links to well-established molecular targets within key colorectal cancer pathways. These include Wnt/β-catenin, PI3K/AKT/mTOR, MAPK/ERK, TGF-β-driven epithelial–mesenchymal transition, p53-related signaling, and pathways involved in angiogenesis, apoptosis, and cell-cycle control. The consistent involvement of these miRNAs in central regulatory networks provides compelling biological support for their potential use as diagnostic biomarkers in colorectal cancer.

Given the complexity of CRC development, it was expected that the molecular mechanisms responsible for the altered expression of each of the 22 distinct miRNAs would vary among them. Thus, the variety of biological functions represented among the miRNAs also represents the various aspects of tumor biology. Therefore, the biological heterogeneity among the miRNAs may have contributed to the large amount of statistical heterogeneity observed across studies (I^2^ > 85%) and supported the conclusion that circulating miRNAs could serve as a useful biomarker class.

Notably, miRNAs involved in diverse biological functions such as cell proliferation and invasion, angiogenesis and apoptosis, and epigenetic regulation showed comparable diagnostic sensitivity. This conjunction suggests that miRNA dysregulation in colorectal cancer is not confined to a single pathway but instead reflects widespread disruption across multiple key oncogenic processes.

More importantly, the similarity of the pathways involved in the diagnostic signals in miRNA in CRC supports the idea that the diagnostic signals in miRNA are due to underlying biological differences in CRC rather than technical variability. The pathways which were consistently involved in miRNA-derived diagnostic signals in CRC included those involved in the regulation of growth signaling (PI3K/AKT, MAPK), epithelial–mesenchymal transition (ZEB /E-cadherin), epigenetic modification (DNMTs), and cell cycle (e.g., cyclins). Collectively, these findings support the notion that the diagnostic utility of miRNA in plasma in CRC is based on the biologic characteristics of CRC tumors.

### 4.3. Clinical Implications

The findings of this investigation carry meaningful clinical implications for the development of screening strategies in early-onset colorectal cancer, particularly the notably robust diagnostic performance observed—sensitivity of 85.68% and specificity of 85.11%. While these results are encouraging, clinical applicability will require validation in large, prospective studies conducted in true screening populations. Whereas routine colorectal cancer screening is currently not recommended for age group under 50 years, the steadfast rise in EOCRC incidence has exposed critical gaps in early detection. In comparison to colonoscopy, which requires bowel preparation, sedation, and specialized facilities, blood-based miRNA testing represents a minimally invasive, patient-acceptable alternative. This may substantially enhance screening uptake in younger individuals who often do not realize that they are at elevated risk.

Moreover, diagnostic accuracy is preserved within EOCRC-specific subgroups. Notably, the high DOR reported in patients aged below 50 years (DOR 36.98) becomes very relevant given the growing evidence supporting the concept that EOCRC might be a biologically different entity with peculiar molecular patterns and more aggressive pathological features. Showing reliable performance in this age group is particularly important for clinical use, as any screening approach must be validated in the very populations it is designed to target. Subgroup analyses reported here provide quantitative support for clinical relevance in young adults using the miRNA-based assay. From a translational perspective, the findings suggest that further evaluation efforts should prioritize individual miRNAs with robust pooled performance rather than miRNA classes as a whole. MiR-21, in particular, meets several criteria for clinical feasibility, including reproducibility, biological plausibility, and compatibility with standardized qRT-PCR workflows. Importantly, circulating miRNAs might serve better as part of multi-marker diagnostic panels rather than as stand-alone biomarkers. The studies identified miRNAs implicated in heterogeneous and complementary oncogenic pathways; thus, the rational combinations of these molecules could further significantly improve diagnostic performance. Such integrative panels may reach sensitivities above 90% and specificities above 95%, thresholds that are particularly desirable for population-level screening applications. From a practical point of view, clinical feasibility in miRNA testing is further favored by the rapid turnaround time of qRT-PCR quantification, which can be completed within 4–6 h and is already available in most diagnostic laboratories. Furthermore, circulating miRNAs prove notably stable, maintaining integrity across multiple freeze–thaw cycles beyond that realized by protein biomarkers and circulating tumor DNA. This stability enhances the reliability and scalability of miRNA-based assays and supports their implementation across diverse healthcare settings, including those with limited laboratory infrastructure.

Although this review focuses on individual circulating miRNAs with reproducible diagnostic performance, reliance on a limited number of predefined biomarkers may constrain diagnostic sensitivity in biologically heterogeneous diseases such as colorectal cancer. Emerging approaches that integrate extended miRNA expression profiles with machine learning and deep learning algorithms offer the potential to capture complex, non-linear patterns that are not detectable using single-biomarker strategies [67,68]. Such data-driven models, particularly when applied to high-dimensional miRNA datasets generated by next-generation sequencing, may further enhance diagnostic robustness and enable individualized risk prediction [69]. However, these approaches require large, well-annotated cohorts, rigorous external validation, and standardized analytical pipelines before they can be translated into clinically reliable screening tools.

Beyond detection, the clinical value of circulating miRNA biomarkers may be enhanced by integration with clinically implementable risk stratification tools. Recent work has demonstrated that phenotype-based clinical scores, such as the Meta-Lung Score developed for colorectal cancer patients with lung-limited metastatic disease, can effectively stratify prognosis and guide management decisions using readily available clinical variables. While such tools address post-diagnostic decision-making rather than screening, they provide a useful translational framework illustrating how molecular biomarkers could be coupled with clinical risk models to support more personalized CRC care pathways following early detection, without detracting from the primary diagnostic focus of the present study [70].

Within the broader colorectal cancer liquid biopsy ecosystem, circulating miRNAs should be viewed as complementary rather than competing biomarkers alongside circulating tumor DNA (ctDNA), DNA methylation markers, and extracellular vesicle-based assays. While ctDNA and methylation signatures offer high tumor specificity, their sensitivity in early-stage or low-burden disease may be limited, particularly in screening settings [71,72]. In contrast, circulating miRNAs represent biologically amplified signals that reflect tumor–host interactions and systemic responses, supporting their utility in early detection contexts [73]. These characteristics favor a multi-analyte strategy in which miRNA-based assays serve as an accessible first-tier screening or triage tool, followed by more tumor-specific modalities for confirmatory testing. Such layered approaches, exemplified by emerging multi-cancer and CRC liquid biopsy platforms, illustrate a pragmatic pathway toward clinical-grade assays that balance feasibility, cost, and analytical rigor [74,75].

### 4.4. Comparison with Existing Literature

This meta-analysis supports and expands previous systematic reviews, providing new information that is important to understand the EOCRC. One meta-analysis by T. Liu et al., published in 2020, investigated circulating miRNAs for CRC in different age groups and showed a pooled sensitivity of 78% and specificity of 82% [57]. These values are slightly lower than those of the present study, with a sensitivity of 85.68% and specificity of 85.11%. The improved performance could indicate a focus on individual miRNAs; this may decrease the methodological variability, and the inclusion of newer studies (2019–2025) may apply more modern analytical methods. Moreover, the current analysis targeted EOCRC populations where biomarker performance is higher because of the aggressive tumor biology of these cases.

Another investigation in 2019, systematically reviewed by Rapado-González et al., proposed circulating miRNAs as promising candidates for colorectal cancer screening. However, the authors also emphasized considerable heterogeneity across studies, largely resulting from differences in study design and the use of non-uniform reference standards [76]. The present meta-analysis addresses these limitations by applying strict inclusion criteria, relying on histopathological confirmation, conducting a thorough QUADAS-2 quality assessment, and performing detailed subgroup analyses. Remarkably, the analysis indicated that geographic region exerted a minimal effect on diagnostic performance (*p* = 0.12), contrasting with prior assertions that ethnic differences substantially influence miRNA utility. This pattern implies that dysregulation of circulating miRNAs may constitute a shared molecular signature across varied populations.

Considering this fact, contemporary American Cancer Society guidelines have increased the age level at which CRC screening should be initiated from 50 to 45 years. Despite this, screening uptake remains poor in the 45–49-year-old age group due to perceived risk and a variety of barriers to procedures. The diagnostic performance observed in the current study would suggest that blood-based miRNA testing could be a viable initial screening strategy in younger populations. A positive test result could prompt a confirmatory colonoscopy, establishing a two-tiered strategy that may maximize resource utilization while maintaining population-level sensitivity.

### 4.5. Limitations

There are several significant limitations in this meta-analysis that require consideration while interpreting the results. First, the methodological quality of included studies was potentially less than optimum. According to the QUADAS-2 assessment, a high or unclear risk of bias was observed in the patient selection domain and the index test domain in 87.5% of studies. Most of these concerns arise from the retrospective nature of the study designs, the reliance on convenience sampling, inadequate blinding, and the lack of established diagnostic thresholds. Only one study met the criteria for an overall low risk of bias, underscoring the prevalence of methodological weaknesses in the current literature. Although sensitivity analyses restricted to higher-quality studies yielded results consistent with the main analysis, the small number of rigorously designed investigations limits the strength of inference and highlights the need for prospective studies conducted in accordance with STARD recommendations.

Secondly, all the studies reviewed under the current analyses used retrospective case–control designs. Although this approach is common in early biomarker research, it can lead to an overestimation of diagnostic accuracy due to spectrum bias. In most cases, patients had already established or advanced-stage disease, while control groups typically included healthy individuals instead of patients with benign colorectal conditions. Additionally, case–control studies do not allow for reliable estimation of positive or negative predictive values, which depend on disease prevalence and are crucial for assessing the true effectiveness of a screening test.

Third, the geographic and ethnic representation of the included studies was limited but still holds the potential of being advantageous in clinical settings. Most studies were conducted in China (62.5%) or Egypt (37.5%), with no representation from Europe, North America, or South America. This distribution may raise concerns about generalizability, particularly given that the most pronounced increases in EOCRC incidence have been reported in Western populations. The absence of data from these regions may restrict the applicability of the findings to populations where improved early detection is most urgently needed. Future multi-cultural and multi-ethnic validation studies are essential to determine whether the observed diagnostic performance of circulating miRNAs is consistent across diverse genetic and environmental backgrounds.

Fourth, substantial heterogeneity was observed across studies, with an I^2^ value of 87.3%, indicating marked inconsistency in diagnostic performance. This variability persisted even after subgroup analyses, suggesting that additional unmeasured factors such as differences in tumor stage, anatomical location, molecular subtypes, sample processing, or laboratory protocols may have influenced the results. Consequently, while pooled estimates provide an overall summary, they should be interpreted cautiously. Greater standardization of study design and laboratory methods will be necessary to reduce heterogeneity and improve comparability across future investigations.

Finally, the absence of standardized normalization strategies for quantifying circulating miRNA is still a major technical constraint. The studies included in this analysis used a mix of different methods, such as endogenous reference miRNAs like miR-16, miR-191, and let-7a, exogenous spike-in controls like cel-miR-39, and various global normalization techniques. While sensitivity analyses focusing on studies that employed validated endogenous controls showed results similar to the overall findings, indicating some level of reliability, this inconsistency makes it tough to directly compare results across different studies. It is crucial to prioritize the establishment of consensus normalization protocols through collaborative, multi-center initiatives to push forward clinical applications. Sensitivity analyses restricted to studies with explicit EOCRC stratification or clear age reporting yielded consistent results, strengthening confidence in the EOCRC-specific diagnostic performance of circulating miRNAs. An additional limitation relates to reference standard verification among control groups. While colorectal cancer cases were uniformly confirmed by histopathology and/or colonoscopy, not all studies applied colonoscopy to control participants. In some studies, controls were defined as healthy individuals or patients with benign conditions based on clinical assessment. This may have introduced misclassification bias and should be considered when interpreting pooled specificity estimates. Future diagnostic studies should ensure uniform colonoscopic verification of control populations to strengthen reference standard validity. Lack of standardization in normalization controls, cut-off definitions, and pre-analytical handling across studies limits direct comparability and underscores the need for harmonized protocols in future validation studies. All included studies relied on targeted qRT-PCR-based miRNA quantification; however, next-generation sequencing approaches may enable more comprehensive and unbiased miRNA discovery and validation, and ultra-non-invasive biofluids such as exhaled breath condensate have been shown to support NGS-based miRNA profiling with good concordance to plasma, highlighting their potential for future screening-oriented applications [77].

Importantly, challenges related to pre-analytical variability, normalization strategy selection, and inter-laboratory reproducibility are not unique to colorectal cancer, but are widely recognized across circulating miRNA research in multiple disease contexts. Emerging work in exhaled breath condensate-based miRNA detection across non-CRC fields demonstrates the technical feasibility of ultra-non-invasive miRNA profiling using next-generation sequencing, while simultaneously underscoring the sensitivity of results to pre-analytical handling and analytical pipelines. Collectively, these cross-field observations reinforce both the promise of novel biofluids and the urgent need for harmonized, MIQE-like guidelines tailored to circulating and extracellular miRNA biomarker studies.

Despite these limitations, the biological plausibility supporting circulating miRNAs as biomarkers for EOCRC remains strong. The miRNAs discussed in this meta-analysis are not just statistically linked to the disease; they are also connected to crucial pathways in colorectal cancer through targets that have been experimentally validated, functional tests, and pathway enrichment analyses. Importantly, the observed diagnostic signals appear to reflect underlying tumor biology rather than technical artifacts alone. The consistency of results across sensitivity analyses, the absence of detectable publication bias, and the reproducibility of findings for well-characterized miRNAs collectively strengthen confidence in their relevance. Nevertheless, these findings should be viewed as a foundation rather than definitive evidence. Large-scale, prospective validation studies conducted in true screening settings are still required before circulating miRNA assays can be responsibly integrated into routine clinical practice for early-onset colorectal cancer detection. Because multiple miRNA evaluations were derived from the same underlying study populations, pooled estimates should be interpreted as performance ranges across biomarkers rather than independent replication across cohorts.

### 4.6. Future Directions

Translating circulating miRNAs into clinical settings for early detection of early-onset colorectal cancer will require progress on several interconnected fronts. One of the most immediate needs is greater consistency in how samples are handled before analysis. Differences in blood collection, processing times, storage conditions, RNA extraction methods, and qRT-PCR workflows continue to introduce avoidable variability across studies. Wider adoption of MIQE-compliant pre-analytical and analytical procedures would substantially improve comparability between datasets. Coordinated international efforts, potentially led by existing initiatives such as the Early Detection Research Network, could play an important role in establishing shared protocols, similar to the standardization that has already been achieved for other molecular biomarkers.

Equally important is the shift from retrospective analyses toward well-designed prospective validation studies. While retrospective case–control cohorts have been valuable for initial discovery, they cannot fully capture how biomarkers perform in real-world screening settings. Large, multicenter studies with predefined cut-off values and harmonized methodologies are needed to confirm diagnostic accuracy in asymptomatic populations. Embedding nested case–control analyses within established prospective cohorts, such as PLCO or the UK Biobank, would allow assessment of miRNA performance in samples collected years before clinical diagnosis. Such designs are particularly relevant for evaluating early detection potential, temporal stability, and true predictive value. Accounting for real-world factors, including test refusal and non-adherence, will also be necessary to generate realistic estimates of population-level effectiveness.

Another promising direction lies in moving beyond single-marker approaches. Circulating miRNAs capture only one layer of tumor biology, and their diagnostic value may be enhanced when interpreted alongside other molecular signals. Integrating miRNA profiles with complementary data such as cell-free DNA methylation patterns, circulating proteins, metabolomic signatures, or gut microbiome features may provide a more complete picture of early tumor development. Advances in machine learning and data integration now make it feasible to explore such multi-omics models, with the potential to identify biomarker combinations that outperform any individual marker alone.

Population diversity also remains a critical gap in the current evidence base. Most existing studies have been conducted in relatively homogeneous cohorts, making it difficult to determine whether observed diagnostic performance is consistent across different ethnic and genetic backgrounds. Dedicated validation studies in diverse populations are needed to assess generalizability and, where appropriate, to define population-specific reference ranges or cut-off values. International collaboration will be essential to address this limitation and ensure its potential relevance for future screening applications.

For circulating miRNAs to move beyond the research setting, attention must also turn to assay development and regulatory pathways. Robust, reproducible, and scalable commercial platforms will be required, supported by strict quality control and regulatory oversight. Partnerships between academic groups and the diagnostics industry could accelerate the development of automated, high-throughput assays that demonstrate not only analytical reliability but also clear clinical validity and utility, in line with regulatory standards such as those set by the FDA and CLIA.

Finally, any proposed screening strategy must be evaluated in terms of cost and feasibility. Comparative cost-effectiveness analyses that examine miRNA-based testing alongside established approaches such as colonoscopy, fecal immunochemical testing, and multitarget stool DNA assays will be essential, particularly in younger populations where screening policies are still evolving. These analyses should incorporate realistic assumptions about test costs, participation rates, downstream procedures, and healthcare resource utilization to provide meaningful guidance for clinicians and policymakers.

## 5. Conclusions

This systematic review and meta-analysis provides strong evidence that individual circulating microRNAs have promising accuracy signals for detecting early-onset colorectal cancer. In EOCRC patients (<50 years), circulating miRNAs demonstrate promising accuracy signals, with pooled sensitivity of 84.4% and specificity of 85.7%. These biomarkers consistently prove effective in diagnosing colorectal cancer in people under 50, a group that is seeing a rise in CRC cases. These markers could change non-invasive screening for younger populations since miRNA dysregulation is linked to key pathways that contribute to cancer development. The functional overlap of various miRNAs that regulate vital tumor processes like growth, invasion, angiogenesis, cell death, and gene regulation highlights their biological importance and assures us that they reflect the fundamental biology of the tumor. However, significant methodological challenges remain. Issues such as retrospective study designs, geographic limitations, a lack of standardization, and substantial variation emphasize the need for more prospective validation studies with strict quality control before clinical implementation. Blood-based miRNA testing offers a promising way to meet the urgent demand for accessible screening methods in early-onset colorectal cancer populations. To achieve this potential, a coordinated multi-center initiative is crucial. This initiative should establish standardized procedures, validate accuracy across diverse groups, integrate multi-omics data, and prove cost-effectiveness. Among evaluated biomarkers, miRNA-specific pooled estimates, particularly for miR-21, represent the most clinically actionable findings of this meta-analysis and should be prioritized for prospective validation in EOCRC screening. Individual circulating miRNAs exhibit promising accuracy signals for early-onset colorectal cancer detection; however, these findings remain hypothesis-generating and require prospective validation in true screening settings before clinical adoption. Successfully completing these tasks would lead to earlier detection and possibly reduce the impact of this increasingly common condition among younger individuals.

## Figures and Tables

**Table 1:** Summary of cut-offs, normalization strategies, and sample processing across included studies.

Study	miRNA	Biofluid	Detection Method	Normalization/Reference	Cut-off Definition	Cut-off Value	Notes
Han L 2021 [32]	miR-15b; miR-16; miR-21; miR-31 (exosomal)	Serum (exosomes)	RT-qPCR	U6	ROC analysis (Youden index)	Not explicitly stated	Panel model evaluated; miR-15b best single marker
Hishida 2022 [34]	miR-26a-5p; miR-223-3p	Serum	RT-qPCR (after microarray)	Not clearly specified	ROC analysis	Not specified	Pre-clinical detection up to 2 years before diagnosis
Karabulut 2024 [35]	let-7; miR-125b; miR-30a	Serum	qRT-PCR	Not specified	ROC analysis	Not specified	miR-30a showed strongest diagnostic value
Li G 2020 [40]	miR-21; miR-210	Serum	qRT-PCR	U6	ROC analysis	Not specified	Also evaluated pre- vs post-surgery levels
Liu X 2019 [29]	miR-1290; miR-320d	Plasma	qRT-PCR	miR-16	ROC analysis	Not specified	Training and validation cohorts used
Pan 2020 [39]	miR-592	Serum	RT-qPCR	U6	ROC analysis	Not specified	Differentiated early CRC and advanced adenoma
Sabry 2019 [37]	miR-210; miR-21; miR-126	Serum	qRT-PCR	U6	ROC analysis	Not specified	miR-210 and miR-21 showed highest sensitivity/specificity
Salah 2020 [30]	miR-1246; miR-23a; miR-451	Serum	qRT-PCR	SNORD68	ROC analysis	Not specified	miR-1246: 100% sensitivity, 80% specificity
Shi 2020 [41]	miR-92a-1	Serum	qRT-PCR	U6	ROC analysis	Not specified	High AUC (0.914)
Shiosaki 2020 [38]	miR-21; miR-29a; miR-92a	Serum (vesicular & non-vesicular)	qRT-PCR	miR-16	Ratio-based ROC analysis	Not specified	Multi-ethnic cohort
Wang 2020 [31]	miR-378e	Serum	RT-qPCR	U6	ROC analysis	Not specified	Combined with LI-cadherin
Wu 2021 [33]	miR-92a	Tissue (not biofluid)	qRT-PCR	U6	ROC analysis	Not applicable	Included for comparison; not circulating
Zhao YJ 2019 [27]	miR-150-5p; miR-99b-5p (exosomal)	Serum (exosomes)	RT-qPCR	cel-miR-39	ROC analysis	Not specified	Early CRC discrimination
Zhao DY 2022 [28]	miR-627-5p; miR-199a-5p	Serum	qRT-PCR	miR-16	ROC + logistic model	Not specified	Integrated model improved AUC
Cui 2021 [36]	miR-1539 (serum & exosomal)	Serum; exosomes	RT-qPCR	U6	ROC analysis	Not specified	Higher specificity for left-sided CRC
Ghareib 2019 [26]	miR-21	Serum	qRT-PCR	U6	ROC analysis	Not specified	Sensitivity 95.8%, specificity 91.7%

Abbreviations: ROC, receiver operating characteristic. The observed heterogeneity in normalization approaches (e.g., miR-16, cel-miR-39, U6), cut-off selection, and sample handling represents a key methodological limitation and likely contributes to between-study variability in diagnostic performance.

**Table 2:** Meta-regression of study-level covariates on diagnostic odds ratio.

Covariate Tested	Effect Estimate (Direction)	*p*-Value
EOCRC status (<50 vs. mixed/late-onset)	Not significant (EOCRC showed balanced sensitivity/specificity but did not explain heterogeneity)	>0.05
Sample size (≥50 vs. <50 CRC cases)	Not significant	>0.05
Geographic region (Egypt vs. China/others)	Numerically higher DOR in Egyptian studies, but not statistically significant	0.12
Biospecimen type (serum vs. plasma)	Not significant; overlapping confidence intervals	>0.10
Detection method (qRT-PCR normalization strategy)	Not significant	>0.05
Study quality (QUADAS-2 risk of bias)	Not significant	>0.05

**Table 3:** Relevant miRNAs and their potential targets.

miRNA	Classification	Target Genes/Pathways	Validation Status	Key References	Accuracy	Critical Notes
miR-21 [42]	Oncogenic	PTEN, PDCD4; Wnt/β-catenin, PI3K/AKT	Extensively validated	Gan et al., 2024	Excellent	Well-established oncogene in CRC
miR-145 [43,44]	Tumor suppressor	c-Myc, KRAS	Extensively validated	Sachdeva et al., 2009; Pagliuca et al., 2013	Excellent	p53-regulated; coordinates with miR-143
miR-200c [45,46]	Tumor suppressor	ZEB1, ZEB2; EMT	Extensively validated	Hur et al., 2013; Prabhakaran et al., 2026	Excellent	Core EMT regulator; prevents metastasis
miR-29a [47,48]	Tumor suppressor	DNMT3A, DNMT3B	Extensively validated	Fabbri et al., 2007; Mo & Cao, 2023	Excellent	Landmark DNMT-targeting studies
miR-92a [49]	Oncogenic	PTEN, p57	Mostly validated	Zhou, 2016	Good	PTEN confirmed; p57 needs verification
miR-1246 [50,51]	Oncogenic	CCNG2; Cell cycle	Extensively validated	Wang et al., 2021; Aalami et al., 2024	Excellent	miR-1246/CCNG2 axis well documented
miR-451 [52,53]	Tumor suppressor	MIF	Extensively validated	Bandres et al., 2009; Bai et al., 2019	Excellent	Direct MIF targeting confirmed
miR-199a-5p [54,55]	Tumor suppressor	HIF-1α	Extensively validated	Zhu et al., 2018; Wang et al., 2017	Excellent	Inhibits angiogenesis

**Table 4:** GRADE Summary of Findings for circulating miRNAs in EOCRC detection.

Outcome	No. of Studies	No. of Participants	Pooled Estimate (95% CI)	Certainty of Evidence (GRADE)	Reasons for Rating
Sensitivity (overall CRC)	16	909 CRC/691 controls	~0.86 (range across studies)	Low ⬤⬤◯◯	Serious heterogeneity; retrospective case–control design; indirectness to screening populations
Specificity (overall CRC)	16	909 CRC/691 controls	~0.85 (range across studies)	Low ⬤⬤◯◯	Serious heterogeneity; risk of bias in patient selection; indirectness
Sensitivity (EOCRC < 50 years)	4	EOCRC subset	~0.84 (range across studies)	Very low ⬤◯◯◯	Small number of studies; imprecision; heterogeneity; indirectness
Specificity (EOCRC < 50 years)	4	EOCRC subset	~0.86 (range across studies)	Very low ⬤◯◯◯	Small sample size; retrospective design; heterogeneity
miR-21 (most validated miRNA)	5	CRC/controls	Favorable accuracy across studies	Low ⬤⬤◯◯	Consistent signal but limited prospective validation; indirectness

The GRADE approach for diagnostic accuracy studies was applied considering risk of bias (QUADAS-2), inconsistency, indirectness, imprecision, and publication bias. Pooled estimates are interpreted as performance ranges rather than definitive test accuracy.

## Data Availability

The original contributions presented in the study are included in the article. Further inquiries can be directed to the corresponding author. The data sets used during the study are available from the corresponding author on reasonable request.

## Supplementary Materials

The following supporting information can be downloaded at https://www.mdpi.com/article/10.3390/cancers18050720/s1: Table S1. Detailed search strategy; Table S2. Basic Characteristics
