# Supplementary material for: Evaluating MiRNAs in Blood-Based Liquid Biopsy for Early-Onset Colorectal Cancer Detection: A Systematic Review and Meta-Analysis
Source: Cancers (Basel). 2026 Feb 24;18(5):720. doi: 10.3390/cancers18050720 (PMC12985209; doi:10.3390/cancers18050720)
Supplement: Supplementary file 1 [file cancers-18-00720-s001.zip › cancers-4113569-supplementary.pdf]

“Evaluating miRNAs in Blood-Based Liquid Biopsy for Early Onset Colorectal Cancer Detection: A Systematic Review and Meta Analysis”

Table S1. Detailed search strategy

Database	Keywords	Results
PubMed	("microRNA" OR "miRNA" OR "mir" OR "microRNAs" OR "miRNAs")	222,472
	("biomarker" OR "biomarkers" OR "diagnostic marker" OR "molecular marker")	927,326
	(“colorectal cancer” OR “CRC” OR “colon cancer” OR “rectal cancer”)	66,276
	#1 AND #2 AND #3	117
Web of Science	colorectal cancer OR CRC OR colon cancer OR rectal cancer AND biomarker OR biomarkers OR diagnostic marker OR molecular marker AND microRNA OR miRNA OR mir OR microRNAs OR miRNAs	422
Embase	colorectal cancer OR CRC OR colon cancer OR rectal cancer AND biomarker OR biomarkers OR diagnostic marker OR molecular marker AND microRNA OR miRNA OR mir OR microRNAs OR miRNAs	232
Cochrane Library	colorectal cancer OR CRC OR colon cancer OR rectal cancer AND biomarker OR biomarkers OR diagnostic marker OR molecular marker AND microRNA OR miRNA OR mir OR microRNAs OR miRNAs	12
Scopus	colorectal cancer OR CRC OR colon cancer OR rectal cancer AND biomarker OR biomarkers OR diagnostic marker OR molecular marker AND microRNA OR miRNA OR mir OR microRNAs OR miRNAs	323
CBD2/CRC-EBD Biomarker Databases	colorectal cancer OR CRC OR colon cancer OR rectal cancer AND biomarker OR biomarkers OR diagnostic marker OR molecular marker AND microRNA OR miRNA OR mir OR microRNAs OR miRNAs	16
Grey Literature (ProQuest, OpenGrey, bioRxiv)	colorectal cancer OR CRC OR colon cancer OR rectal cancer AND biomarker OR biomarkers OR diagnostic marker OR molecular marker AND microRNA OR miRNA OR mir OR microRNAs OR miRNAs	22

Table S2. Basic Characteristics

**Study**	**Year**	**Country of the Study**	**Study Design**	**Sample Size**		**Reported Age (Years)**	**EOCRC < 50**	**Sample Type**	**Method**	**Single/ Panel**	**miRNA**	**AUC**	**Sensitivity**	**Specificity**	**Validation**	**TP**	**FN**	**FP**	**TN**
				**HC**	**CRC**														
Ghareib	2020	Egypt	Case-Control	48	48	55.2 ± 7.9 (Mean ± SD)	Yes	Serum	qRT-PCR	Single	miR-21	0.94	95.8	91.7	No	46	2	4	44
Sabry 1	2019	Egypt	Case-Control	101	35	51.97 ± 12.18 Mean ± SD	Yes	Serum	qRT-PCR	Single	miR-210	0.93	88.6	90.1	No	31	4	10	91
		Egypt									miR-21	0.97	91.4	95		32	3	5	96
		Egypt									miR-126	0.67	88.6	50.5		31	4	50	51
Shi	2020	China	Case-Control	68	148	Below and above 60	Not specified	Serum	qRT-PCR	Single	miR-92a-1	0.91	81.8	95.6	No	121	27	3	65
Shiosaki	2020	Hawaii (USA) and Japan	Case-Control	18	73	18 to 89 (Age range)	Yes	Serum	qRT-PCR	Single/ Panel	miR-21	0.76	72.6	70.6	No	53	20	5	13
Salah 1	2020	Egypt	Case-Control	30	37	49.1 ± 16.42 Mean ± SD	Yes	Serum	qRT-PCR	Single	miR-1246	0.92	100	80	No	37	0	6	24
											miR-451	0.76	73	80		27	10	6	24
Li 1	2020	China	Case-Control	20	40	Below and above 60	Not specified	Serum	qRT-PCR	Single	miR-21	0.86	88.9	83.3	No	36	4	3	17
											miR-210	0.81	88.9	72.2		36	4	6	14
Zhao 1	2022	China	Case-Control	30	60	66.95 ± 10.52 Mean ± SD	No	Serum	qRT-PCR	Single/ Panel	miR-627-5p	0.97	87	100	HC = 33, CRC = 20	52	8	0	30
											miR-199a-5p	0.9	93	70		56	4	9	21
Hishida	2022	Japan	Case-Control	7	7	58.4 ± 6.9 Mean ± SD	No	Serum	qRT-PCR	Single	miR-26a-5p	0.84	100	60	8 HC, 8 CRC	7	0	3	4
Pan	2020	China	Case-Control	50	84	Below and above 60	Not specified	Serum	qRT-PCR	Single	miR-592	0.88	86.6	73.4		73	11	13	37
Karabulut 1	2024	Turkey	Case-Control	20	60	60 (31–81)Mean (Age range)	Yes	Serum	qRT-PCR	Single	let-7	0.76	70	70		42	18	6	14
											miR-125b	0.76	70	65		42	18	7	13

											miR-30a	0.939	93	75		56	4	5	15
Han 1	2021	China	Case-Control	150	117	51.60 ± 11.41 Mean ± SD	Yes	Serum	qRT-PCR	Single/ Panel	miR-15b	0.86	81.33	91.8	CRC = 80, HC = 67 HC	95	22	12	138
											miR-21	0.75	95.06	94.44		111	6	8	142
											miR-31	0.75	91.95	97.62		108	9	4	146
Wu	2021	China	Case-Control	60	164	32–75 Age range	Yes	Serum	qRT-PCR	Single	miR-192-5p	0.84	84.6	79.2		139	25	12	48
YJ 1	2019	China	Case-Control	153	165	Below and above 61	Not specified	Serum	qRT-PCR	Single	miR-99b-5p	0.63	32.1	90.8	Yes	53	112	14	139
											miR-150-5p	0.71	75.2	58.8		124	41	63	90
Cui	2020	China	Case-Control	49	51	58.92 ± 11.39 Mean ± SD	Yes	Serum	qRT-PCR	Single	miR-1539	0.65	38	96.6	In tissues	19	32	2	47
Wang	2020	China	Case-Control	90	110	62.13 ± 9.21 Mean ± SD	No	Serum	qRT-PCR	Single/ Panel	miR-378e	0.93	89	80		98	12	18	72
Liu	2019	China	Case-Control	15	15	Below and above 60	Not specified	Plasma	qRT-PCR	Single/ Panel	miR-1290	0.96	78.79	93.33	CRC = 80, CRA = 50, HC = 30	12	3	1	14
											miR-320d	0.89	93.94	73.33		14	1	4	11
